# Sub-5 nm Silicon Nanopore Sensors: Scalable Fabrication via Self-Limiting Metal-Assisted Chemical Etching

**DOI:** 10.1021/acsami.4c19750

**Published:** 2025-01-30

**Authors:** Fabio De Ferrari, Shyamprasad N. Raja, Anna Herland, Frank Niklaus, Göran Stemme

**Affiliations:** † Division of Micro and Nanosystems, KTH Royal Institute of Technology, Malvinas väg 10, Stockholm 100 44, Sweden; ‡ Division of Nanobiotechnology, SciLifeLab, Department of Protein Science, KTH Royal Institute of Technology, Tomtebodavägen 23a, Solna 171 65, Sweden; § AIMES - Center for Integrated Medical and Engineering Science, Department of Neuroscience, Karolinska Institute, Stockholm 171 77, Sweden

**Keywords:** nanopores, sensing, nanofluidic devices, MACE, DNA translocation

## Abstract

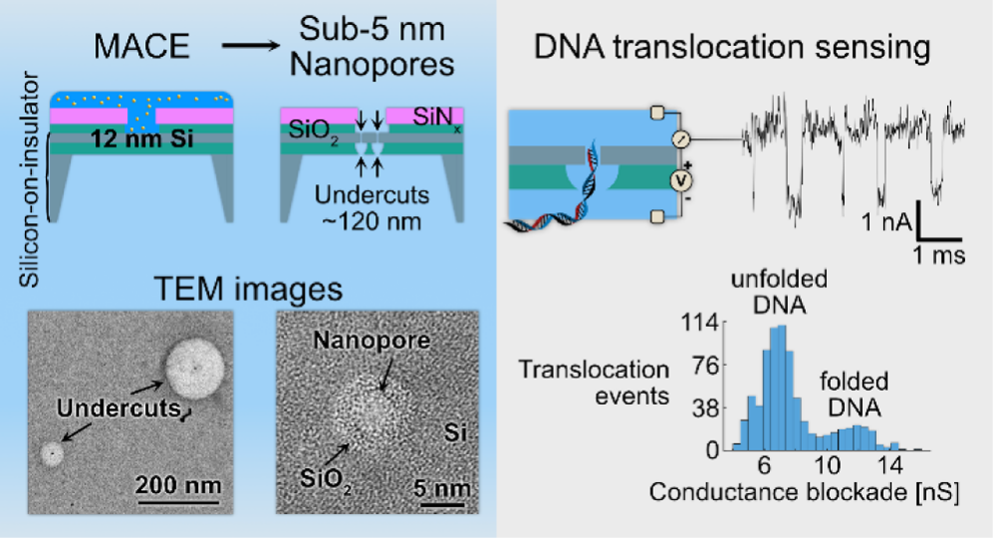

Solid-state nanopores offer unique possibilities for biomolecule sensing; however, scalable production of sub-5 nm pores with precise diameter control remains a manufacturing challenge. In this work, we developed a scalable method to fabricate sub-5 nm nanopores in silicon (Si) nanomembranes through metal-assisted chemical etching (MACE) using gold nanoparticles. Notably, we present a previously unreported self-limiting effect that enables sub-5 nm nanopore formation from both 10 and 40 nm nanoparticles in the 12 nm thick monocrystalline device layer of a silicon-on-insulator substrate. This effect reveals distinctive etching dynamics in ultrathin Si nanomembranes, enabling precise control over nanopore dimensions. The resulting nanopore sensor, suspended over self-aligned spheroidal oxide undercuts with diameters of just a few hundred nanometers, exhibited low electrical noise and high stability due to encapsulation within dielectric layers. In DNA translocation experiments, our nanopore platform could distinguish folded and unfolded DNA conformations and maintained stable baseline conductance for up to 6 h, demonstrating both sensitivity and robustness. Our scalable nanopore fabrication method is compatible with wafer-level and batch processing and holds promise for advancing biomolecular sensing and analysis.

## Introduction

Solid-state nanopores have become a versatile tool for bioanalysis with single-molecule resolution, enabling studies of various analytes, from single nucleotides and DNA oligomers to proteins and protein–DNA complexes.^1^ Furthermore, they have been adopted as readout sensors for DNA-based digital information storage.^2^ Beyond proof-of-concept demonstrations with synthetic analytes, solid-state nanopore sensing applications have recently advanced to include clinically relevant applications, such as RNA conformational dynamics,^3^ biomarker quantification,^4^ and single nucleotide mutations.^5^ Fabrication of sub-5 nm diameter solid-state nanopores is essential for high-resolution sensing and sequencing, particularly for applications involving proteins, such as sodium dodecyl sulfate denatured proteins^6−8^ with typical diameters of approximately 1–3 nm.^6,9^ In nanopore sensing, optimal signal-to-noise is achieved when the nanopore is only slightly larger than the diameter of the analyte, and high resolution along biomolecular strands is typically attained by using ultrathin membranes (∼10 nm or less).^9−12^

Sub-5 nm solid-state nanopores are fabricated primarily through serial techniques, including TEM-drilling,^10^ FIB-drilling,^11^ laser-assisted photothermal etching,^12^ quartz capillaries pulling,^2^ and controlled dielectric breakdown.^13^ Recently, plasmonic-assisted photochemical etching has also been demonstrated to create sub-5 nm pores through controlled metal nucleation inside metallic nanorings.^14^ While these methods achieve excellent pore-size control and can be used to fabricate nanopores in a variety of solid-state materials, they lack scalability and cannot produce nanopores with precisely defined diameters using wafer-scale manufacturing processes. Even extreme ultraviolet (EUV), the highest resolution lithography technology currently used in state-of-the art commercial semiconductor manufacturing, is limited to ∼10–20 nm features due to optical and chemical shot noise limitations.^15^ While top-down lithographic methods struggle to reach dimensions relevant for biomolecule sensing, bottom-up chemical synthesis approaches have succeeded at sub-5 nm scales, such as fabrication of nanoparticles and quantum dots.^16^ In silicon (Si) nanostructuring, bottom-up synthesis has facilitated the production of high-density Si nanowire arrays,^17^ nanoporous Si, and black Si photoabsorbers, all made possible by metal-assisted chemical etching (MACE).^18,19^

MACE—a self-limiting redox process that etches Si only in contact with metals such as gold or silver in the presence of hydrogen peroxide (H_2_O_2_) and hydrofluoric acid (HF)—offers scalability for patterning dense nanostructures. The shape and size of the etched features can be controlled by adjusting the dimensions of the used metal nanoparticles, making MACE attractive for scalable, high-density nanostructuring. However, achieving isolated features smaller than 5 nm with precise control remains challenging, and reports on isolated, sub-5 nm nanopores are limited.^20^ Previous studies have demonstrated the utility of MACE in creating arrays of nanopores much larger than 10 nm in Si membranes that are >100 nm thick for realizing high-aspect-ratio nanochannels.^21,22^ However, many emerging biomolecule sensing applications demand scalable manufacturing of ultrathin membranes (thickness ∼10 nm or less) with single or a few sub-5 nm diameter nanopores to achieve high-resolution detection and sequencing.

To address this gap, we present a scalable method for manufacturing sub-5 nm nanopores by utilizing Au nanoparticles with well-defined diameters,^23^ and controlled deposition density on the Si surface.^24^ By integrating a newly discovered self-limiting etching mechanism with a refined MACE process, we created a Si-based sensor based on rapid, scalable production of sub-5 nm nanopores. Implementing this approach on a silicon-on-insulator (SOI) substrate, with a thin monocrystalline Si layer as the nanopore membrane, allows us to achieve a low-noise, mechanically stable nanopore platform. Noise reduction is achieved through three key mechanisms: first, isolating the Si device layer from the Si handle layer using the buried oxide (BOX) layer, which decreases parasitic capacitances;^25^ second, minimizing the suspended membrane area to a diameter of below 300 nm by HF etching the BOX layer through the nanopore, thereby decreasing effective capacitance and enhancing stability; and third, protecting the top Si surface with a silicon oxide (SiO_2_) – silicon nitride (SiN_*x*_) insulator stack, leaving only a few 2 μm circular openings that limit Si contact with the analyte.^26^

Notably, our SOI platform produced nanopores significantly smaller than the employed nanoparticles—a surprising outcome that diverged from the expected match between pore and particle diameters. Leveraging this discovery, we fabricated sub-5 nm nanopores using both 10 and 40 nm Au nanoparticles in a 12 nm thick Si membrane. Using these devices, we performed proof-of-concept DNA translocation experiments to validate the platform’s capabilities for biomolecule sensing. Our results show performance comparable to existing sensors, while the platform’s scalability and rapid production make it a promising tool for advanced bioanalytical applications.

## Results and Discussion

**Figure 1:**
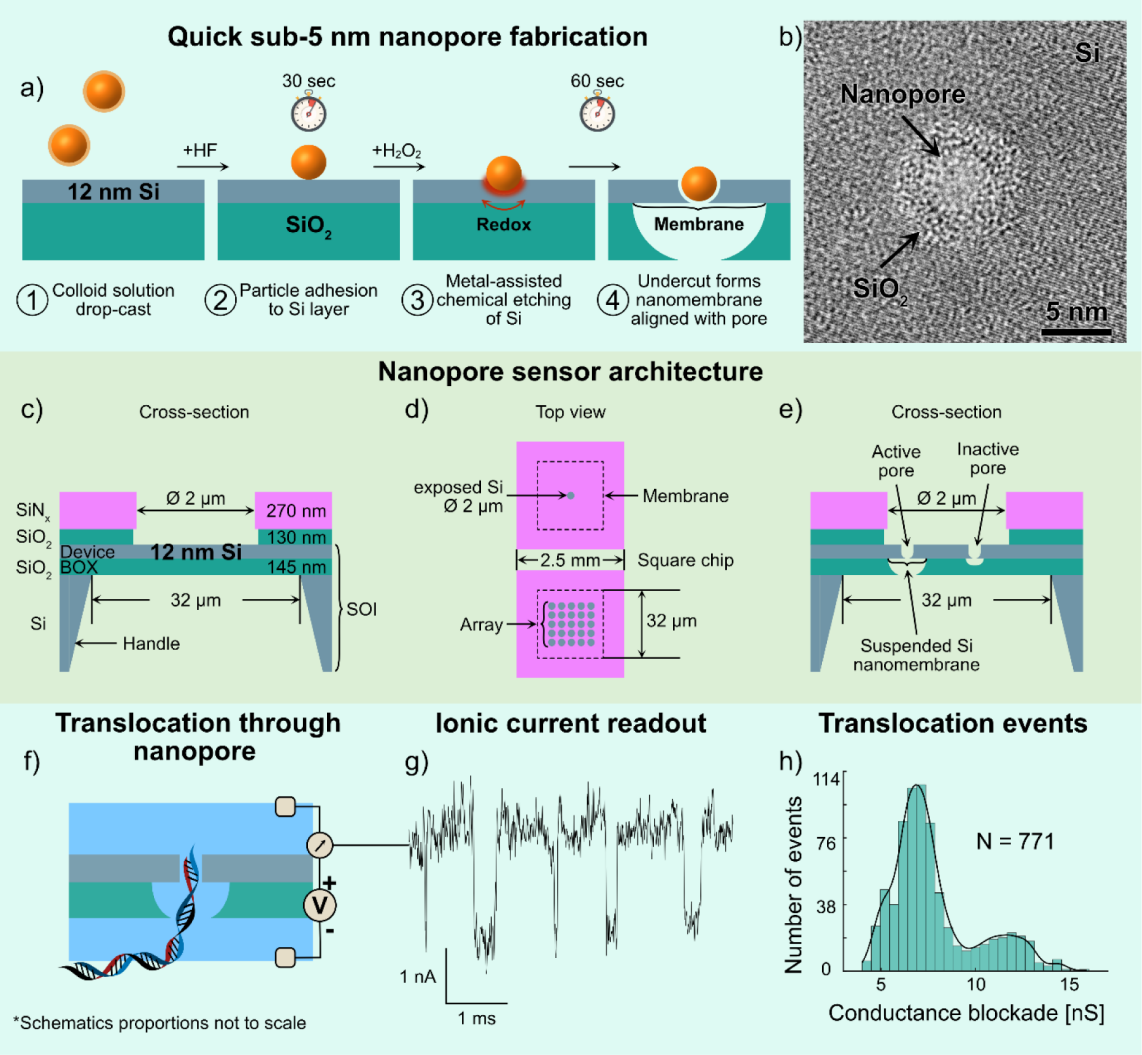
Nanopore fabrication approach using MACE, nanopore sensor architecture, and demonstration of DNA sensing. (a) Schematic of the nanopore fabrication process: (1) an aqueous solution of monodisperse citrate-capped gold (Au) nanoparticles is drop-cast onto a bare monocrystalline silicon (Si) surface. (2) The addition of hydrofluoric acid (HF) neutralizes the citrate capping, inducing precipitation and nanoparticle deposition on the Si surface. (3) The addition of hydrogen peroxide (H_2_O_2_) initiates the metal-assisted chemical etching of the Si. This localized redox reaction occurs at the Si–Au interface, where the etching proceeds perpendicular to the <100> Si surface, and the Au nanoparticle burrows into the Si layer. (4) When the particle etches through the 12 nm thick Si layer, the HF starts to etch the silicon oxide (SiO_2_) layer below, forming a self-aligned undercut around the nanopore and creating a suspended Si nanomembrane. A water rinse is used to stop the reactions. (b) Bright-field TEM image of a Si nanopore after Au nanoparticle etching. The MACE-etched lumen shows a ∼4 nm diameter nanopore surrounded by a ∼9 nm diameter amorphous SiO_2_ area. Arrows indicate the outer edges of the pore and the oxide area. (c) Cross-sectional schematic of the processed SOI substrate that is used for the nanopore fabrication. The thin Si device layer is supported by the 145 nm thick buried SiO_2_ (BOX) layer of the SOI substrate. A dielectric passivation stack (SiO_2_ and SiN_*x*_) with patterned micron-scale circular openings is fabricated on top of the Si device layer. The membranes are formed in the SOI substrates by locally removing the Si handle layer on the backside of the SOI substrate. (d) Precise definition of the membrane areas in which the nanopore can form is achieved by patterning openings in the dielectric passivation on top of the Si membrane layer. One single 2 μm diameter circular opening, or an array with 5 × 5 of such openings defines the Si membrane area in which etching of nanopores can happen. Chip dimensions: 2.5 mm × 2.5 mm. (e) An active nanopore is produced when its undercut in the BOX layer extends through its entire thickness, resulting in a Si nanomembrane suspended over a ∼250 nm diameter circular area centered around the nanopore. Pores with shallow (not through-etched) undercuts in the BOX layer do not contribute to the ionic current signals. (f) Schematic of double-stranded DNA sensing experiments using nanopore sensor devices. (g) Concatenated events trace showing ionic current blockades generated from DNA translocation events. (h) Conductance blockade distribution, demonstrating the ability of the nanopore sensor platform to detect DNA translocations.

To form nanopores in 12 nm thick Si membranes we used metal-assisted chemical etching (MACE) with Au nanoparticles of a precise size in solution (Figure 1a). For this process we drop-cast the Au nanoparticle solution onto the membrane that consists of a stack of a 12 nm thick Si layer on top of a 145 nm thick SiO_2_ layer (BOX) that are part of a SOI substrate. The addition of liquid HF caused the Au particles to adhere to the Si surface. After 30 s, H_2_O_2_ was introduced to initiate the MACE process. The resulting anisotropic etching of the Si in the immediate vicinity of the Au particles proceeds perpendicular to the <100> Si surface toward the Si-BOX interface. When the etching front reaches the Si-BOX interface, nanopore formation is complete, and the HF present in the solution starts etching the BOX layer through the nanopore, creating undercuts with diameters of a few hundred nanometers surrounding the nanopores. Initially, we selected 10 nm Au nanoparticles, anticipating formation of nanopores of a similar diameter to the nanoparticles. Interestingly, bright-field transmission electron microscopy (BF-TEM) imaging revealed that the resulting nanopores had diameters of below 5 nm (Figure 1b), indicating that pores much smaller than the diameter of the nanoparticles had been produced.

The membranes were fabricated with ∼30 μm × 30 μm square openings, featuring a 12 nm thick Si device layer sandwiched between dielectric layers, i.e., a 145 nm thick BOX layer below, and a layer stack of 130 nm thick SiO_2_ and 270 nm thick SiN_*x*_ above (Figures 1c and S1). Nanopore formation was controlled statistically through selective exposure of Si areas via precisely defined openings in the SiN_*x*_/SiO_2_ layers, enabling selective Au nanoparticle contact with the Si surface to initiate MACE (Figure 1c). We developed two distinct membrane architectures: one featuring a single 2 μm diameter opening and another incorporating a 5 × 5 array of 2 μm openings (Figure 1d). This approach enabled tuning of nanopore density balancing the need for efficient DNA capture while maintaining minimal total pore count for enhanced sensing capability. The etching procedure is outlined in the Methods section, and Supplementary Note 1 provides an in-depth description of our custom MACE protocol, examining how each process parameter influences the final density of nanopores per membrane.

We fabricated the membranes on 2.5 × 2.5 mm^2^ chips containing one membrane and performed subsequent MACE processes at the chip scale. The MACE duration after adding H_2_O_2_ was empirically set to produce nanopores with undercuts fully etched through the entire thickness of the BOX layer, which is essential for creating an active nanopore (Figure 1e). To validate our platform, we conducted dsDNA translocation experiments (Figure 1f). Ionic current recordings across the membrane showed characteristic current blockades corresponding to dsDNA translocation events (Figure 1g). The conductance drop histogram revealed two distinct peaks around 7 nS and 12 nS, which we attribute to unfolded and folded DNA conformations, respectively (Figure 1h). These results demonstrate the ability of our nanopore sensors to detect and differentiate between folded and unfolded DNA conformation during translocation.

**Figure 2:**
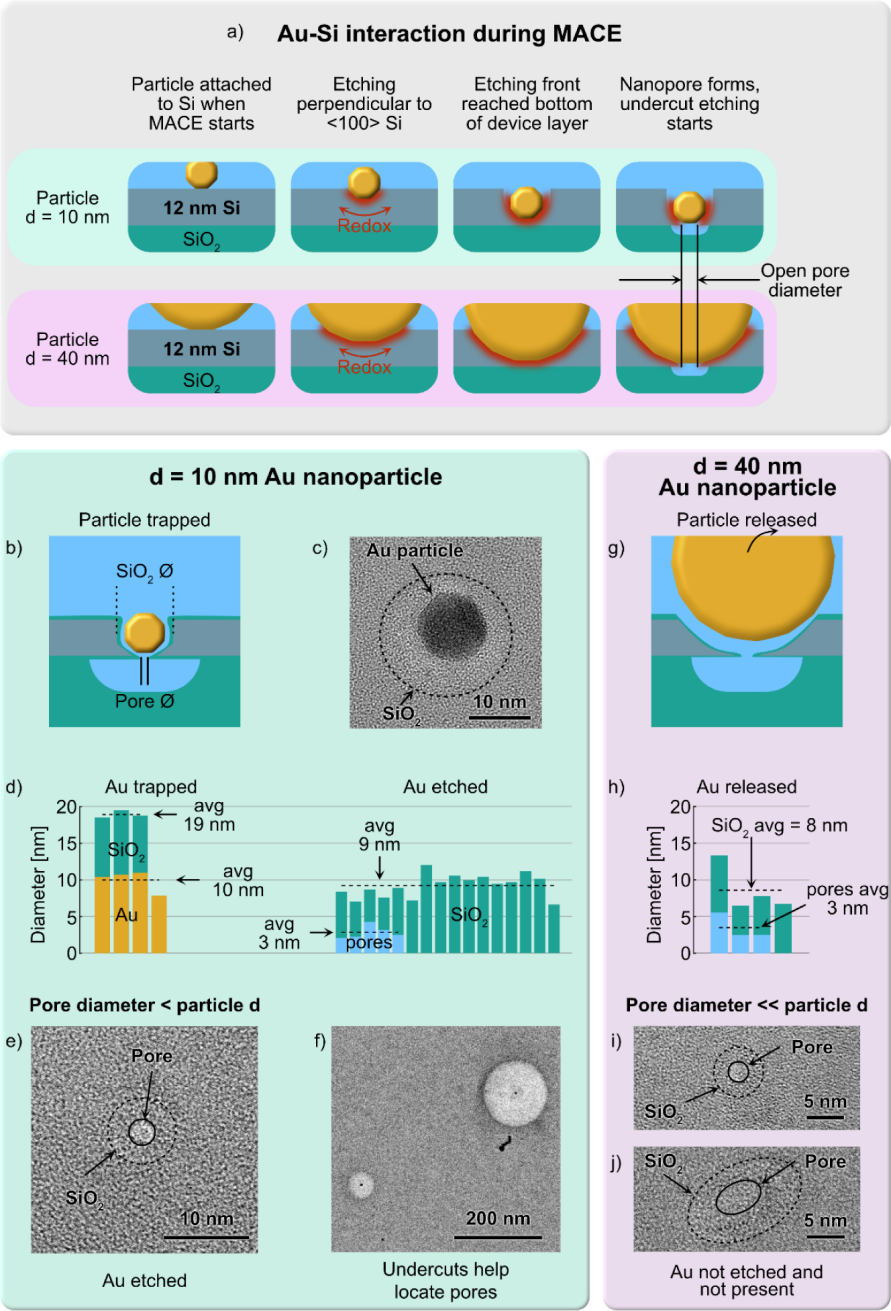
Effects of Au nanoparticle size on nanopore morphology. (a) Schematic of the MACE process on an SOI substrate: A redox etching front forms at the Au–Si interface, driving the nanoparticle perpendicular to the Si device layer surface. Etching automatically stops at the Si-BOX layer interface. Ten nm particles become fully embedded in the 12 nm Si layer; 40 nm particles remain mostly outside. A nanopore smaller than the nanoparticle forms at the Si-BOX interface. All schematics represent the nominally spherical particles as regular polygons to highlight the faceted geometry of colloidal nanoparticles. (b) Cross-sectional schematic of a 10 nm Au nanoparticle trapped in the nanopore after rinsing. Dashed lines: outer edge of SiO_2_ area; solid line: open nanopore diameter. (c) BF-TEM image of a trapped 10 nm Au particle surrounded by amorphous SiO_2_, showing nonconcentric SiO_2_ area and faceted particle edges. (d) Measured diameters of SiO_2_ areas, Au particles, and nanopores. Left, Au trapped: average Au particle diameter 10 nm, average SiO_2_ area diameter 19 nm. Right, Au etched after MACE: average SiO_2_ area 9 nm, average open nanopore diameter 3 nm. (e) TEM image after Au particle etching shows nanopore smaller than the initial particle diameter. Solid circle: open nanopore; dashed circle: outer edge of SiO_2_ area. (f) Self-aligned undercuts in BOX layer below Si device layer, which aid in locating nanopores during TEM imaging. TEM image shows 10 nm Au particles centered in the undercuts. (g) Schematic of a 40 nm Au nanoparticle released from 12 nm Si layer after MACE. (h) Measured nanopore diameters formed using 40 nm Au particles: average SiO_2_ area diameter 8 nm, average open nanopore diameter 3 nm. (i) BF-TEM image of a ∼3 nm diameter nanopore where the 40 nm Au nanoparticle is absent after MACE. (j) BF-TEM image of noncircular pore resulting from 40 nm Au nanoparticle.

Bright-field transmission electron microscopy (BF-TEM) and energy-filtered transmission electron microscopy (EF-TEM) were employed to characterize the morphology of the fabricated nanopores. We found that nanopores produced by MACE using 10 and 40 nm Au nanoparticles exhibited similar diameters, despite the 4-fold difference in particle size (Figure 2a). This observation indicates a self-limiting effect of the MACE process in our SOI devices, which hinders the Au nanoparticles from crossing the Si-BOX layer interface, resulting in the formation of nanopores with diameters significantly smaller than the catalyzing nanoparticles. To explore our hypothesis of hindered penetration of the nanoparticles at the Si layer and stopping of the MACE process at the Si-BOX layer interface, we conducted additional TEM analysis of nanopores. In samples prepared with 10 nm Au nanoparticles, we observed a nanoparticle at the center of each successfully formed nanopore, as indicated by the presence of an easily identifiable undercut (Figure 2f). These nanoparticles appeared trapped within the Si device layer (Figure 2b) in the lumen of the nanopore they had formed. Surrounding each Au nanoparticle was an annular amorphous area (Figure 2c), likely SiO_2_ formed from post-MACE Si oxidation in air, with an average diameter of 19.0 ± 0.5 nm (*N* = 3) (Figure 2d, left). This amorphous region was encircled by monocrystalline Si. In 3 out of 16 cases, the SiO_2_–Si boundary appeared visually blurred, necessitating the use of Fast Fourier Transform (FFT) filtered images to measure the amorphous area diameter (detailed procedure in Figure S4, additional TEM images in Figure S5).

Our investigation of the nanopore formation process continued with the preparation of samples where the 10 nm diameter Au nanoparticles were etched away using a potassium iodide (KI/I_2_) solution within a minute after performing MACE. In these samples, the outer diameter of the SiO_2_ area around the nanopores averaged 9.2 ± 1.5 nm (*N* = 16, Figure 2d, right), significantly smaller than the 19.0 ± 0.5 nm observed in sample where the Au nanoparticles were not removed after MACE. Additionally, in 5 out of 16 samples, a small open pore area with an average diameter of 2.8 ± 0.9 nm was observed (Figure 2d, right). These dimensions were further validated in an independent set of devices fabricated using 10 nm Au nanoparticles, where TEM analysis revealed an average pore diameter of 2.8 ± 0.8 nm (*N* = 11), demonstrating consistent formation of sub-5 nm pores across different fabrication batches. The remaining 11 samples of the set of 16 samples showed no clearly identifiable open pores in TEM in the center of the undercut but only an amorphous inclusion in the crystalline Si (Figure S5, bottom row). The absence of visible pores in these samples suggests that the nanopores formed during MACE were so small that native oxide formation and carbon deposition during TEM imaging led to the pores appearing closed. It is important to note that the pore sidewalls are expected to be crystalline Si immediately after MACE, and the Si begins to oxidize during rinsing in deionized water and continue during air storage. However, native SiO_2_ formation is self-limiting, stopping at a thickness of ∼2 nm. In a nanopore geometry, this native SiO_2_ growth on the Si sidewalls results in nanopore diameter shrinkage.^27^ Even when accounting for the growth of a 2 nm thick native SiO_2_ layer, the diameter of the resulting pores were significantly smaller than expected based on the diameter of the Au nanoparticles (manufacturer specification: 10 ± 2 nm;^28^ TEM measurement:10 ± 1 nm, *N* = 4, Figure 2d). If even half of the 10 nm particle had crossed the Si-BOX layer interface, the resulting open pore area after native SiO_2_ formation would have been significantly larger, around 6–10 nm in diameter. These observations suggest that the MACE of Si stopped and further Au nanoparticle motion into the Si device layer ceased when the MACE etching front broke through the Si device layer and reached the BOX layer (Figure 2a). Furthermore, the much larger size of the amorphous SiO_2_ area when the Au nanoparticle was left in place, compared to when it was etched immediately after MACE (Figure 2d), indicates continued oxidation of Si near the embedded Au nanoparticle. Moreover, we note that when post-MACE Au etching was not performed, we created an Au nanoparticle trap, with 10 nm diameter Au nanoparticles captured in the nanopore channel they created in 12 nm thick Si, accessible to fluid flow from both membrane sides (Figure 2b, c). This configuration offers a unique opportunity to study individual Au nanoparticles trapped in a nanometer-thin Si layer.

Our experiments on nanopore formation using MACE with 10 nm Au nanoparticles revealed that nanopores form with much smaller diameters than the nanoparticles themselves as the etching front reaches the Si-BOX interface. To confirm this theory, we used 40 nm Au nanoparticles and found, consistent with our hypothesis, that the open pore diameters were in the sub-5 nm range (3.5 ± 1.7 nm, *N* = 3, Figure 2h), much smaller than the 40 nm diameter of the nanoparticles.

TEM imaging further revealed an amorphous SiO_2_ region surrounding the nanopores with an outer diameter of only 8 ± 3 nm (*N* = 4), significantly smaller than expected based on the particle size. We attribute this difference to the faceted geometry of the Au nanoparticles observed in TEM images. This geometry locally reduces the contact area between the nanoparticle and Si surface during MACE, creating a much smaller pore than an idealized spherical shape would produce.

Upon examining the centers of undercuts where nanopores form, we observed that 40 nm Au nanoparticles were absent despite no additional Au etching after MACE (Figure 2i,j). In contrast, 10 nm particles remained trapped, likely due to size differences: while 10 nm particles are confined within the 12 nm thick Si device layer, most of the 40 nm particle’s volume remains outside (Figure 2a, lower panel), allowing it to rinse away after pore formation (Figure 2g).

To further explore the effect of particle size, we evaluated the etching process with 10, 40, and 100 nm particles. Our investigation revealed that only a fraction of deposited particles formed complete nanopores, while others created shallow indentations on the Si surface (Figure S6). When the etching front reached the Si-BOX interface, particle behavior was size-dependent: particles smaller than 12 nm (10 nm) became trapped in the nanopore, while larger particles (40 and 100 nm) were released. This size-dependent behavior suggests that the SiO_2_ etching reaction at the Si-BOX interface reduces Au–Si adhesion, facilitating the release of larger particles during rinsing, whereas 10 nm particles remain trapped due to their smaller size.

To confirm the role of the Si-BOX interface in nanopore formation, we conducted MACE on both bulk Si and SOI substrates with a 12 nm device layer. In bulk Si, particles formed pores slightly larger than their own size (Figure S8). However, 40 nm particles on SOI substrates created undercuts without detectable nanopores, likely due to the sub-10 nm size (Figure S9). Similarly, 100 nm particles showed large undercuts without visible nanopores (Figure S10), underscoring the unique, self-limiting MACE etching behavior in thin Si layers supported by a BOX layer.

**Figure 3:**
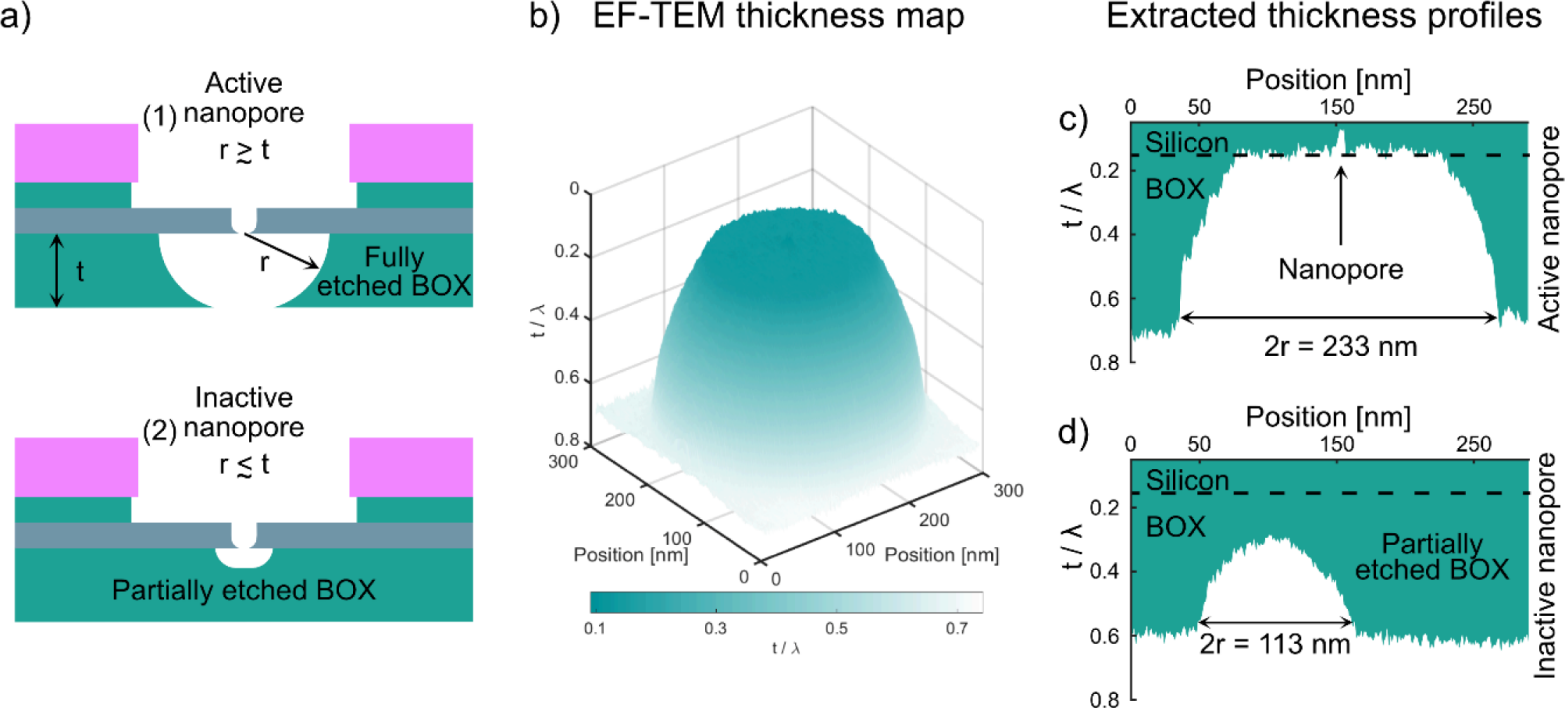
EF-TEM analysis of fully and partially etched undercuts in the BOX below the Si layer surrounding the nanopores. (a) A functional ionic current sensor requires the BOX layer undercut to extend fully through its thickness (1). In our devices, this necessitates an undercut radius exceeding ∼115 nm (80% of the BOX layer thickness). Smaller undercuts result in incomplete BOX etching, preventing ionic current flow through the nanopore (2). b) EF-TEM 3D map of an active nanopore type (1) sample, showing the relative thickness (*t*/λ) of the Si and the BOX layers and the surrounding undercut. (c) Extracted thickness profile from the diagonal cross-section of the EF-TEM map in (b) for a type (1) sample with the Si membrane and a central nanopore (relative thickness approaching zero). The diameter of the undercut in the BOX below the Si layer is 233 nm. (d) Extracted thickness profile for a type (2) sample with a partially removed BOX layer featuring a semicircular undercut profile with a diameter of 113 nm. The line profiles represent four-pixel averages along the diagonal through the membrane center.

To gain additional insights into nanopore formation and BOX layer etching, we created thickness maps of the nanomembrane area surrounding our nanopores using energy-filtered TEM (EF-TEM). This technique offers cross-sectional profile information by computing a relative thickness map (*t*/λ) from unfiltered and zero-loss images using the Poisson statistics of inelastic scattering: . Here the ratio between the thickness t and the mean free path (mfp) λ is calculated from the ratio of the total unfiltered intensity *I*_t_ and the zero-loss filtered intensity I_0._^29^ Our EF-TEM analysis aimed to confirm that nanopores in the Si layer with undercuts in the BOX layer of radii that are much smaller than the BOX layer thickness contain residual SiO_2_ and do not represent open pores, rendering them unsuitable for sensing (Figure 3a). Therefore, we examined two pore sample types: (1) pores with expected complete BOX removal on the backside of the Si layer surrounding the pore (Figure 3a, upper panel) and (2) pores with only partially removed BOX on the backside of the Si layer surrounding the pore (Figure 3a, lower panel). We extracted cross sections, including the nanomembrane center, where the nanopore was expected, from the 3D thickness maps (Figure 3b). The t/λ line profile from fully open undercuts revealed typical rounded edges of the remaining BOX, formed by isotropic SiO_2_ etching and a local thickness decrease at the membrane center indicated the presence of a sub-10 nm nanopore in a type (1) sample (Figure 3c). In contrast, the cross-section extracted from a type (2) sample with a smaller undercut displayed significantly higher thickness values, suggesting residual BOX (Figure 3d). These EF-TEM analyses demonstrate the isotropic etching of the BOX through the MACE-formed nanopore and emphasize the necessity for complete BOX removal during the etching process. This establishes a lower bound for the undercut diameter required in the BOX layer below the Si layer to form nanopores suitable for sensing applications. Additional details about the thickness mapping are reported in Supplementary Note 2 and the mapping of a nanopore is shown in Figure S11. An added feature of the etched BOX undercuts, measuring hundreds of nanometers in size, is that they greatly facilitate the identification of the nanopore location in TEM and SEM images. In bright-field TEM images, the undercut areas appeared as circular regions of light contrast (Figure 2f), while in SEM images, these areas were quickly identified as regions of dark contrast (Figure S6).

**Figure 4:**
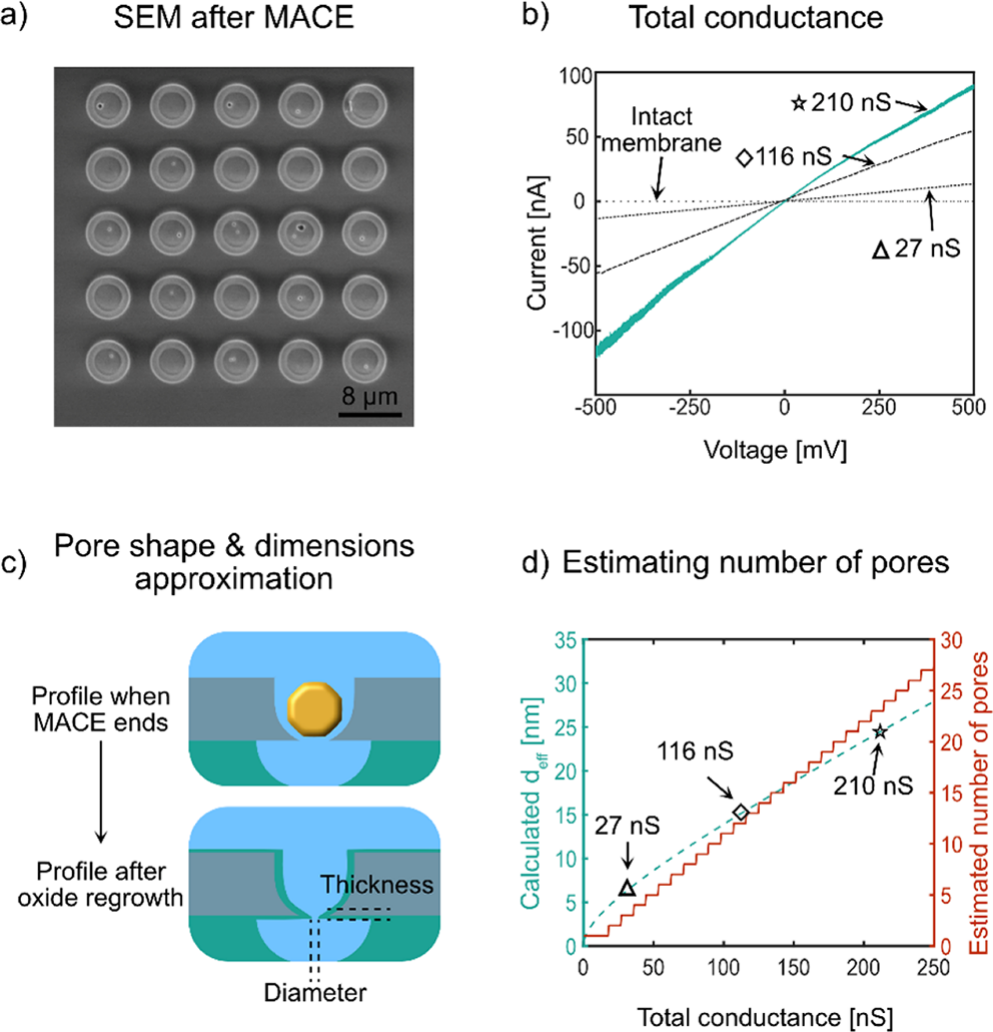
Electrical characterization of nanopore devices and estimation of pore dimensions. (a) SEM image of the nanopore chip consisting of one membrane with a 5 × 5 array of circular openings in the SiN_*x*_/SiO_2_ passivation layers on top of the Si layer, defining the Si areas on the membrane where one or more active nanopores formed during the MACE process. The exposed Si area in each circular opening increased from a diameter of 2 μm before etching to about 4 μm after etching. (b) Current–voltage (*I*–*V*) sweeps in 1 M KCl recorded from three independent nanopore chips made using 10 nm Au nanoparticles in the 12 nm thick Si layer, each containing an unknown number of nanopores (triangle, diamond, and star), and *I*–*V* sweep from a chip with a membrane that does not contain any nanopore (intact membrane). (c) Schematic of the nanopore dimensions considered during estimation from electrical measurements: diameter 3 nm and length 7 nm. (d) Estimated effective single pore diameter (*d*_eff_) and number of nanopores producing the total conductance for the three evaluated nanopore chips in (b). The approximate number of pores per membrane was calculated assuming pore dimensions corresponding to the dimensions described in (c).

To allow electrical characterization of the fabricated nanopores and to detect DNA translocations, precise control over nanopore density is crucial. Therefore, at least one active nanopore per membrane is required. We adopted a combination of statistical analysis and empirical testing to produce samples with about 10 pores on a membrane containing a 5 × 5 array of circular openings. This strategy increased the likelihood of producing functioning devices, albeit at the cost of reduced likelihood of producing sensors with a single nanopore. We only used 10 nm Au nanoparticles for nanopore sensor fabrication due to their higher nanoparticle concentration in stock solution compared to the 40 nm particles.^23^ This higher concentration allowed for higher nanopore yield. Given the sub-5 nm size of the fabricated pores, direct visualization via SEM was not feasible. Instead, we used the number of undercuts in the SOI layer visible in SEM images as a proxy for the pore count on a membrane (Figure 4a). This approach provides a reliable indication of pore formation during process recipe development while circumventing the resolution limitations of SEM imaging for direct pore observation. We conducted a series of electrical characterizations to evaluate the performance of the nanopores sensors that were produced using 10 nm Au particles. We characterized the ionic conductance of the nanopores by performing electrical measurements in 1 M potassium chloride (KCl) solution. We found that the current–voltage (*I*–*V*) characteristics were typically ohmic with minimal hysteresis. Figure 4b shows the *I*–*V* curves of three selected nanopore samples produced under identical conditions, displaying a range of conductances (G) from 27 nS to 210 nS, alongside the *I*–*V* curve of an intact membrane (where MACE was not performed) for reference.

To verify the integrity of the measured nanopore membranes and establish a lower bound for the membrane leakage current we performed additional control experiments. Therefore, we measured the *I*–*V* characteristics of a chip with a membrane that does not contain any nanopore, i.e., where MACE was not performed. These measurements exhibited capacitive hysteresis and current values below 30 pA. The *I*–*V* curve remained unchanged both when removing the top SiO_2_ with HF and when exposing the membrane to the complete MACE solution without gold nanoparticles, confirming that gold particles are essential for nanopore formation (Figure S7). Current increased to about 80 pA only when removing the BOX layer on the backside, exposing the entire 900 μm^2^ Si area to the KCl solution during measurements (Figure S7). In all these control experiments, the maximum measured current was still more than 2 orders of magnitude smaller than the conductance of chips containing 3 nm pores. These experiments confirmed that membranes without pores displayed electrical behavior distinct from membranes with open nanopores, validating the absence of significant conductive defects in the membrane caused by MACE. Furthermore, the experiments confirmed the importance of the presence of the BOX layer for modulating the electrical properties of the membrane and reducing the leakage current.

The shape and dimensions of a nanopore play a crucial role in its electrical characteristics. Due to practical limitations, samples that were measured electrically could not be imaged in SEM afterward. Therefore, we could not count the number of undercuts in these samples to determine the number of nanopores that contributed to the total conductance measured. To estimate an upper bound for the number of pores based on the total measured conductance, we adopted the single pore diameter of 3 nm observed in our TEM analysis and a pore length of about 7 nm estimated from our DNA translocation experiments, as discussed in detail later in this section (Figure 4c). It is important to note that while this shape approximation gives us a reasonable estimate, the actual pore sizes may vary slightly due to factors such as native oxide formation or interactions with the KCl solution during measurements. To relate the pore geometry to its electrical characteristics, we used the well-known cylindrical nanopore model,^30,31^ which relates the pore diameter (*d*), pore length (*L*), and ionic conductance (*G*): . The solution conductivity (σ) was measured to be 11.8 S/m, and the conductance (*G*) was obtained from the slope of the linear fit of the *I*–*V* characteristics. From this equation, we can calculate the conductance of a single nanopore, approximately 9 nS, and use it to estimate the number of pores present in the membrane (Figure 4d). This estimate implicitly assumes that all pores contributing to the total conductance are identical. For comparison, we also calculated the effective diameter (*d*_eff_) of a hypothetical single nanopore, which produced the total measured conductance (Figure 4d). For the nanopore device with 27 nS conductance, we calculated *d*_eff_ to be 5.8 ± 0.6 nm. While nanoparticle aggregates could affect total conductance measurements, our deposition protocol follows O’Reilly et al.^24^ to minimize aggregation during the particle deposition step. Based on the single isolated pore conductance estimate, this total conductance likely results from three nanopores. The device with 116 nS conductance yielded a *d*_eff_ of 15.5 ± 1.2 nm, corresponding to approximately 13 nanopores. For the device with 210 nS conductance, we calculated a *d*_eff_ of 24.3 ± 1.8 nm, suggesting the presence of about 23. In addition, we performed control experiments to verify the integrity of the measured nanopore membranes and establish a lower bound for the membrane leakage current. Therefore, we measured the *I*–*V* characteristics of a chip with a membrane that does not contain any nanopore, i.e., where MACE was not performed (Figure 4b, intact membrane). These measurements exhibited capacitive hysteresis and conductance of 20 ± 2 pS.

To evaluate pore stability, we compared *I*–*V* sweeps of a nanopore device before and after DNA translocation experiments. The *I*–*V* characteristics remained nearly identical after 6 h of DNA translocation measurements at bias voltages ranging from 100 mV to 400 mV. The initial conductance of 206.0 ± 0.1 nS decreased only slightly to 189.0 ± 0.1 nS (Figure S12), indicating excellent stability of the nanopores. This stability can be attributed to the nature of the pore surface. Our pores are made of Si, which is mechanically stable but features a hydrophobic surface that would be expected to clog over time.^32^ However, as observed in our TEM characterization, the Si surface is naturally coated with a native SiO_2_ layer. This native oxide layer makes the surface hydrophilic, likely minimizing the incidence of clogging and allowing the pore to function for hours with only a slight decrease in conductance.

**Figure 5:**
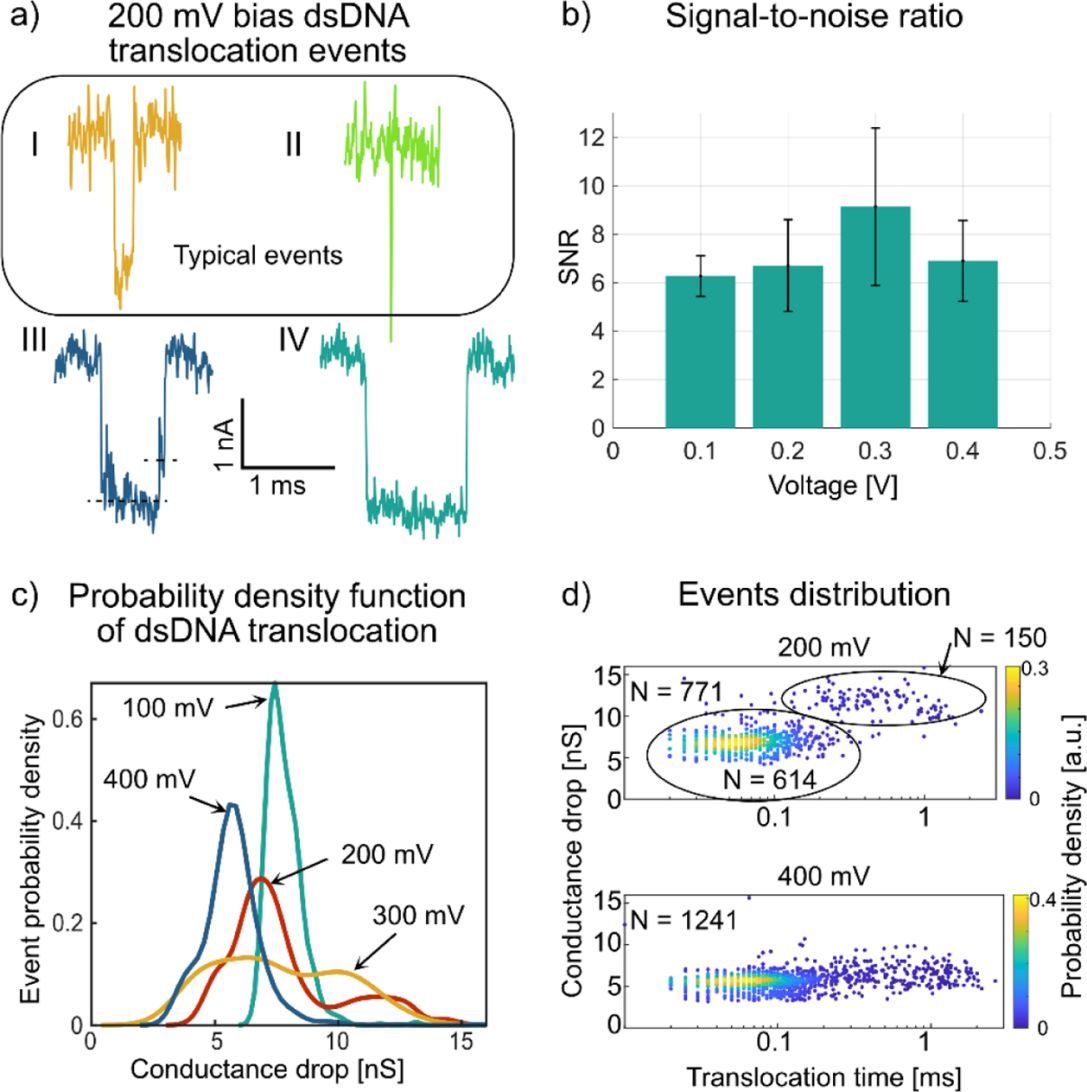
DNA translocation experiments and analysis. Data collected using the nanopore devices featuring the 210 nS conductance in Figure 4, containing multiple nanopores on the membrane. (a) Raw data (200 kHz sampling rate, unfiltered) of four extracted DNA translocation (current blockade) events at a bias voltage of 200 mV, showing examples of typical single-level events with (I) average and (II) short duration of the DNA translocation event, as well as (III) a two-level DNA translocation event and (IV) a DNA translocation event with a long duration. (b) Signal-to-noise ratio (SNR) of unfolded dsDNA translocation events versus applied bias voltage. Error bars indicate standard deviation. The plot illustrates the voltage-dependent signal quality of the nanopore sensing device. (c) Probability density functions of conductance drop (Δ*G*) due to DNA translocation events at different bias voltages (100–400 mV). Two peaks are distinguishable at bias voltages of 200 and 300 mV, whereas at 100 and 400 mV, only one major peak is observed. (d) Scatter plot of conductance drop (Δ*G*) versus DNA translocation time (Δ*t*) of 771 and 1241 translocation events recorded at bias voltages of 200 and 400 mV, respectively. Each point is colored according to the corresponding (Δ*G*, Δ*t*) interval probability. Two clusters of events around conductances of 7 nS and 13 nS at a bias voltage of 200 mV are highlighted, which account for 80% and 19% of the observed events. These two clusters presumably originate from unfolded and folded dsDNA translocations, respectively. The sample set taken at a bias voltage of 400 mV instead shows a higher number of DNA translocation events during the same recording time. However, it shows only one cluster corresponding to unfolded DNA translocation events.

To further validate the functionality of our MACE-fabricated nanopores as sensors, we performed dsDNA translocation experiments using Calf Thymus DNA with a maximum length of 2 kbp. For these experiments, we selected the nanopore device with 210 nS conductance (Figure 4b), as the higher number of pores increased the likelihood of observing translocation events. Following conductance characterization in 1 M KCl, we introduced DNA to the backside of the membrane and recorded translocation events at bias voltages ranging from 100 mV to 400 mV. The event detection rate remained relatively constant over time, with a representative example shown in Figure S13, where 771 events were detected over 10 min, yielding an average interval of 0.8 s between events. Given that most event durations were below 1 ms, we concluded that simultaneous translocations across different pores were highly unlikely. Typical DNA translocation events showed conductance drops between 5 nS and 8 nS, with translocation times of less than 1 ms (Figure 5a, events I and II). Most detected events were single-level, exhibiting only one conductance step below the baseline. However, we also observed a few multilevel events (Figure 5a, III) and events with durations longer than 1 ms (Figure 5a, IV). This sensor’s signal-to-noise ratio (SNR) varied with the applied bias voltage. We computed SNR values for individual events and compared them across different traces and bias levels using the event report generated by EventPro. For recordings performed at a bias voltage of 100 mV, the SNR value was determined to be 6.1 ± 0.6 (*N* = 707). At this voltage, the detection of 707 DNA translocation events necessitated the analysis of three 5 min traces, resulting in a total recording time of 15 min. The SNR improved with increasing bias voltage, reaching a maximum of 7.8 ± 1.9 (*N* = 355) at a bias voltage of 300 mV (Figure 5b). These results indicate that 300 mV represents the optimal measurement condition for the nanopore sensor device, as increasing the bias voltage beyond this value did not yield further improvements in signal quality. Beyond affecting SNR, the applied bias voltage also influenced conductance drops and DNA translocation times, which are critical parameters in characterizing DNA translocation events. We expect that reducing the number of active nanopores in the membrane (possibly down to one) will further improve the SNR as a lower total baseline current also reduces baseline noise.^26^ We found voltage-dependent behavior by analyzing the probability density function of conductance drop for translocation events (Figure 5c). At bias voltages of 100 mV and 400 mV, single peaks of conductance drop were observed at 8 nS and 6 nS, respectively. Notably, at intermediate bias voltages of 200 mV and 300 mV, two distinct peaks emerged: 7 nS and 11 nS at 200 mV and 6 nS and 10 nS at 300 mV. The voltage-dependent changes in peak distribution indicate an interplay between the applied bias voltage and the DNA translocation dynamics.^10,33,34^ At lower bias voltages, such as 100 mV, the electrophoretic forces might not be sufficient to translocate folded DNA fragments efficiently. Additionally, a lower bias voltage diminishes the SNR, making it more challenging to discriminate small variations in conductance drop due to DNA folding. This could explain the observation of a single, broader peak at a bias voltage of 100 mV. As the voltage increased to 200 mV and 300 mV, the improved SNR ratio likely enabled better resolution of folded and unfolded states, resulting in the observed dual peaks. Notably, the SNR increased for increasing bias voltages from 100 mV to 300 mV, facilitating improved event detection and analysis. However, at higher bias voltages (400 mV), the SNR decreased again, potentially due to increased noise, changes in DNA conformation, and faster DNA translocation times. At the highest applied bias voltage of 400 mV, the increased electrophoretic forces might promote entropic stretching of the DNA or alter the proportion of folded versus unfolded translocations,^35^ explaining the transition back to a single peak in the conductance drop distribution. These observations underscore the importance of optimizing the applied bias voltage for a given nanopore device to balance efficient DNA translocation, SNR, and the ability to resolve different DNA conformational states.

A scatter plot of the conductance drop versus DNA translocation time at a bias voltage of 200 mV for 771 detected events revealed two distinct event populations: (1) events with low amplitude and short DNA translocation time, comprising ∼80% of all events, and (2) events with higher amplitude and longer DNA translocation time, comprising ∼19% of all events (Figure 5d, upper panel). Statistical analysis of these populations revealed a significant difference (*p* ≪ 0.001, two-sample *t* test), confirming the robustness of our observations. A similar analysis of 1241 events detected at a bias voltage of 400 mV showed that a larger number of events could be detected during the same recording time at a higher bias voltage. However, only a single event population was discernible at the lower conductance drop, suggesting a decreased sensitivity at higher bias voltages (Figure 5d, lower panel). Moreover, our observed DNA translocation times ranged from tens of microseconds up to a few milliseconds, aligning with values reported in comparable studies.^26,32,36^ We used the conductance drop data from the DNA translocation events to estimate the effective pore length and diameter. Therefore, we employed the equation  to calculate the effective pore length (*L*) from the peak conductance drop (Δ*G*), where σ is the solution conductivity and *d*_DNA_ is the diameter of the DNA molecule.^30,31^ Subsequently, the equation  was used to determine the effective diameter of an equivalent single nanopore (*d*_eff_), substituting *L* with the calculated effective pore length. It should be noted that this calculation provides the diameter of a single pore that would generate the total measured conductance. At the same time, our actual nanopore device likely contains multiple smaller pores. Our analysis of the DNA translocation events detected at 200 mV bias voltage revealed a baseline conductance of 214 ± 5 nS and a conductance drop (Δ*G*) of 6.8 ± 1.1 nS. Assuming a dsDNA diameter of 2.2 nm,^30,31^ we estimate the effective nanopore length to be *L* = 6.6 ± 1.1 nm and the effective diameter of an equivalent single nanopore to be *d*_eff_ = 24.4 ± 1.9 nm. Notably, the calculated pore length is less than the Si layer thickness of 12 nm, as measured by interferometry. We extended this analysis to include two additional nanopore devices that successfully detected DNA translocation events. One device measured at a bias voltage of 400 mV exhibited a baseline conductance of 85 ± 3 nS, with a conductance drop (Δ*G*) of 3.6 ± 0.9 nS and SNR = 6.7 ± 1.1 (*N* = 122). For this device, we calculated an effective pore length of *L* = 12.5 ± 3.1 nm and an effective single pore diameter of *d*_eff_ = 14.9 ± 1.6 nm. The other sample, recorded at a bias voltage of 700 mV, showed a baseline conductance of 93 ± 10 nS and a conductance drop (Δ*G*) of 8.5 ± 1.5 nS and SRN = 3.9 ± 0.4 (*N* = 304), yielding an effective pore length of *L* = 5.3 ± 0.9 nm and an effective single pore diameter of *d*_eff_ = 12.2 ± 1.4 nm. Across all samples, the calculated effective pore lengths were similar to or smaller than the Si device layer thickness. In two out of three samples, the much shorter effective pore length than the Si layer thickness supports the proposed self-limiting MACE mechanism, which sharply narrows the lumen near the Si-BOX interface. This results in nanopores with effective lengths shorter than the total membrane thickness. While the calculated *d*_eff_ is larger than the Au nanoparticles used during MACE, this likely results from several small nanopores, rather than a single large pore.

In summary, our platform demonstrates the capability to detect DNA and distinguish its folding states. By employing DNA as a molecular ruler, we provided additional evidence supporting the partial penetration model, indicating that the Au nanoparticles form nanopores with effective lengths shorter than the Si layer thickness. Our nanopore fabrication approach offers advantages in size control, scalability, and minimal equipment requirements. While currently optimized for chip-scale processing, the protocol using 60 s of MACE shows promise for wafer-level implementation, where hundreds of sensors can be produced simultaneously. However, to achieve this scale further refinement of particle deposition uniformity across large areas is required, as detailed in Supplementary Note 1. The observed self-limiting etching mechanism provides a foundation for reliable formation of sub-5 nm pores that could enable high-throughput sensor production. Furthermore, optimizing the process to achieve single pores on the membranes holds promise for even higher sensitivity. Altogether, this platform has great potential to advance the development of highly sensitive nanopore-based sensors, with great promise for application in biomolecular detection and analysis, biomedical applications, and DNA-based information storage, leading to exciting new research.

## Conclusions

Our study demonstrated the fabrication of sub-5 nm diameter nanopores in Si nanomembranes using MACE. Leveraging commercially available Au nanoparticles and SOI substrates, our approach enables scalable production of sub-5 nm diameter nanopores, much smaller than the catalyzing Au nanoparticles. This was due to the interplay between a self-limiting effect on MACE at the Si-BOX (buried oxide) interface and the nanoparticle geometry, which we discovered in this study. Our fabrication process creates self-aligned hemispherical undercuts in the BOX layer below the Si layer, resulting in a robust nanomembrane structure featuring low electrical noise properties. Electrical characterization and DNA translocation experiments confirmed the platform’s functionality as a biomolecular sensor distinguishing between folded and unfolded DNA conformations. Furthermore, it exhibited good stability during extended DNA translocation experiments, with only slight changes in conductance after up to 6 h of operation. Our nanopore fabrication approach offers advantages in size control, scalability, and minimal equipment requirements. Thus, it will make solid-state nanopores more accessible for a wide range of biomedical applications, opening new avenues for biomolecular detection and analysis.

## Methods

### Fabrication of Si Membranes

SOI wafers (Soitec, France) ⟨100⟩, with a 70 nm thick Si device layer, 145 nm thick BOX layer, and 400 μm thick Si handle layer were used. The Si device layer had p-type boron doping with a maximum resistivity of 11.5 Ohm cm. The wafers were cleaned in piranha solution (3:1 vol/vol mixture of H_2_SO_4_ and H_2_O_2_) for 10 min followed by a 30-s etch in buffered oxide etchant (BOE) (10:1 vol/vol of 40% NH_4_F in water to 49% HF in water) to eliminate the native SiO_2_. An online simulator^37^ was employed to estimate the dry oxidation time needed to achieve the desired thickness of the Si device layer of the SOI wafer, adopting the Massoud model.^38^ All dry oxidations were performed at 950 °C, assuming the absence of native SiO_2_ on the Si surface before oxidation.

Following oxidation, a 270 nm thick layer of SiN_*x*_ was deposited by PECVD (Applied Materials, USA, Precision 5000 Mark II) on top of the Si device layer of the SOI wafer. Blue tape was applied as protection of the deposited SiN_*x*_ layer, while a 3 min etch in BOE removed the SiO_2_ of the handle layer on the backside of the SOI wafer. After proper rinse and drying of the wafer, a 270 nm thick SiN_*x*_ hard mask was deposited by PECVD on the backside of the SOI wafer. Resist MICROPOSIT SPR700–1.2 (Micro Resist Technology GmbH, Germany) was spin-coated on both sides with Maximus 804 (ATMsse, Germany) at 5000 rpm with a 60-s soft bake at 90 °C on a hot plate and patterned using a dose of 175 mJ/cm^2^ on a maskless photolithography system (Heidelberg Instruments GmbH, Germany, MLA150 with “Write Mode I”). Resist development for 50 s in Microposit MF-CD-26 created the opening in the resist, followed by 1 min rinse in deionized water.

Reactive ion etching (Applied Materials, USA, Precision 5000 Mark II) of the SiN_*x*_ mask created openings on the front and back sides of the SOI wafer. Using remover mr-Rem 700 (Micro Resist Technology GmbH), resist residues were stripped from the wafer before mounting the wafer in a wafer holder (Advanced Micromachining Tools GmbH, Germany, AMMT). The holder protected the front side of the SOI wafer, while the handle layer on the backside of the SOI wafer underwent etching in 25% KOH solution at 90 °C. Once the membranes were formed, consisting of the Si device layer and the BOX layer below the Si device layer, resist was spin-coated on the front side of the wafer to protect the surface during dicing (Disco, Germany, DAD3241) of the wafer into individual 2.5 mm × 2.5 mm sized chips, each chip containing a single square 32 μm × 32 μm sized membrane. SEM imaging of a membrane cross section and the layer stack is provided in Figure S1.

### Nanopore Fabrication by MACE

Individual processing of the chips with the completed membranes started with a 10 min resist removal in acetone and a subsequent 10 min cleaning in piranha solution. Blue tape was then applied to the backside of each chip to protect the BOX during the frontside etching steps.

To create the pores, the SiO_2_ in the etched openings of the SiN_*x*_ layer on the Si device layer was removed with a short HF (49%) etch, followed by a DI water rinse. We then drop-cast 4 μL of Au nanoparticle solution (Nanopartz Inc., USA) onto the front surface of the chip. For 10 nm nanoparticles, we used part number A11-10 with a concentration of 4.96 × 10^12^ NPS/mL. For 40 nm nanoparticles, we used part number A11-40 with a concentration of 7.75 × 10^10^ NPS/mL. The manufacturer-specified Au nanoparticle sizes were 10 ± 2 nm^28^ and 40 ± 4 nm,^39^ respectively. While the manufacturer claims a shelf life of up to seven years^23^ for these nanoparticle solutions, our investigation revealed that solution aging affects the resulting particle concentrations on the sample surface and, consequently, the number of pores formed during MACE. A measurable change in nanopore yield was observed after a few months of storage of the nanoparticle solutions, underscoring the importance of regular quality control for this fabrication process.

To tune the density of the fabricated nanopores, several methods were explored. First, decreasing the Au nanoparticle concentration of the solution was considered (first step of Figure 1a). This was primarily implemented by diluting solution samples with a high Au nanoparticle concentration, as it was not practical to increase concentration above the delivered stock solution. Second, extending the HF destabilization time was shown to increase the number of particles sticking to the Si surface^24^ (second step of Figure 1a). However, this approach was generally avoided as the HF also etched the SiN_*x*_ and SiO_2_ passivation layers on the substrate, risking exposure of the entire Si device layer. Lastly, increasing the number of circular openings in the SiN_*x*_ and SiO_2_ passivation layers increased the exposed Si area and thus, the number of pores formed (Figure 1d). Through experimentation, the 5 × 5 array of openings in the SiN_*x*_ and SiO_2_ passivation layers to expose the underlying Si layer was found to be optimal for yielding a few nanopores per membrane. To destabilize the citrate capping and promote particle deposition on the Si surface,^24^ 20 μL of 49% HF was added directly onto the chip where 4 μL of Au nanoparticle solution was drop cast. After 30 s, 4 μL of 30% H_2_O_2_ was introduced to initiate the Si etching process. The DIW/HF/H_2_O_2_ ratio was 14/72/14 v/v/v, adapted from the work of Schneider et al.^40^ Finally, after performing MACE for 60 s, the chip was rinsed in water. Thereafter, the blue tape was removed, and the chip was ready to be mounted between two polydimethylsiloxane (PDMS, SYLGARD 184, USA) adaptors. This MACE protocol was tailored to achieve controlled nanopore formation, with detailed parameter analysis presented in Supplementary Note 1.

It is crucial to note that handling concentrated HF presents significant health and safety risks. Therefore, it is strongly recommended to adhere to proper safety precautions, including the use of personal protective equipment. Additionally, familiarize yourself and your team with emergency procedures and proper waste disposal protocols when working with hazardous chemicals.

### Imaging

For visualization and rapid count of the undercuts in the BOX layer, scanning electron microscopy (SEM) was employed. The samples were imaged using an Ultra 55 SEM (Zeiss, Germany) at an accelerating voltage of 5 keV.

For TEM sample preparation, the Au was removed immediately after MACE using I_2_/KI solution (1 g I_2_, 4 g KI in 40 mL water). These samples are labeled “Au etched” in Figure 2d. An additional HF etching step was performed approximately 18 h before TEM imaging to remove native oxide, which was nevertheless visible during imaging. With our process conditions maintaining ρ = [HF]/([HF] + [H_2_O_2_]) above 88%, minimal porous Si formation is expected.^18^ However, any nanoscale surface roughness from the MACE process could contribute to accelerated native oxide growth compared to native oxide growth on pristine crystalline Si surfaces. Images were captured at 200 keV using a 2100F microscope (JEOL Ltd., Japan). Image analysis was performed using DigitalMicrograph (Gatan Inc., USA) and ImageJ (Rasband, USA) software tools.

In BFTEM images, Si crystal planes are visible when properly aligned with the beam. Even slight tilts can reduce plane visibility. In cases where planes were not immediately apparent, FFT filtering was applied to highlight the Si lattice periodicity, clearly delineating the boundaries between crystalline Si, amorphous SiO_2_, and the pore (detailed procedure in Figure S4).

To measure the diameter of the SiO_2_ area in the TEM images, an oval selection was made, and the diameter was considered as the average value between the oval’s width and height. The same procedure was used to measure pore size when visible. Images used for measurements in Figure 2d and 2h were acquired with a resolution of 0.026 nm/px. An additional set of 11 pores was analyzed using DigitalMicrograph (Gatan) by drawing circular ROIs over each pore in images acquired with a resolution of 0.1 nm/px, with mean and standard deviation calculated from these measurements. Examples of images used to measure Au nanoparticle size, the SiO_2_ areas in samples with and without Au nanoparticles, and open pore areas are shown in Figure S5.

For relative thickness calculation, images for total intensity *I*_t_ and zero-loss filtered intensity *I*_0_ were collected using the same imaging settings. These were corrected for sample drift before computing the relative thickness as  according to standard procedure.^29^

### Electrical Measurements and DNA Translocation

The chip was sealed using Ecoflex 00–35 FAST (Smooth-On, Inc., USA) between two Polydimethylsiloxane (PDMS, SYLGARD 184, USA) adaptors, and mounted in a custom-made flow cell. Ecoflex (EcoFlex 00-35 FAST, Smooth-On Inc., USA) was applied with a blunt needle and the adaptor was placed on the chip, one side at a time, allowing for curing of the Ecoflex before repeating the procedure on the other side of the chip. The flow cell served as the platform for conducting ionic current measurements and was 3D printed with a Form 3 printer (Formlabs, USA) with clear resin and held together with metal screws.^41^

Ionic current measurements were performed in a Faraday cage with the nanopore Reader 100 kHz (Elements SRL, Italy) to minimize electromagnetic noise interference. Two silver wires, pretreated by soaking for 10 min in a 5% bleach (Klorin) solution, established contact between the reader and the flow cell.

DNA samples of 2 kbp dsDNA (UltrePure Calf Thymus DNA Solution, ThermoFisher, USA) were diluted to 20 nM in buffered 1 M KCl and 10 mM HEPES solution (pH 8, conductivity 11.8 S/m). This solution was added to the backside cis chamber of the chip (i.e., the Si handle layer side of the SOI substrate), while the trans chamber contained the same buffered KCl solution without DNA. A positive bias voltage was applied to the frontside trans chamber of the chip (i.e., the Si device layer side of the SOI substrate).

Translocation experiments were performed at bias voltages from 100 mV up to 400 mV and current measurements recorded for 5 min at the time. The sampling rate was set to 200 kHz.

Data analysis employed a combination of custom code in MATLAB (MathWorks, USA) for raw data visualization and plotting, and the Matlab app Event Pro 3.0^42^ for event detection and extraction of dwell times and conductance drops from filtered raw data. Key parameters for Event Pro included a filtering frequency of 20 kHz, Level 2 analysis depth, PDC of 5.5, window size of 0.5 s, and the “Smooth Noisy Data” option for baseline smoothing.

Event Pro generated an analysis report containing detailed information for each detected translocation event, including the signal baseline current and standard deviation. The signal-to-noise ratio (SNR) for each event was calculated by dividing the event baseline current by the baseline signal standard deviation for translocation events identified as unfolded translocations.

## Supporting Information Available

Additional TEM and SEM images; detailed description of custom MACE protocol and energy-filtered TEM analysis (PDF)
