# Supplementary material for: Sub-5 nm Silicon Nanopore Sensors: Scalable Fabrication via Self-Limiting Metal-Assisted Chemical Etching
Source: ACS Appl Mater Interfaces. 2025 Jan 30;17(6):9047–58. doi: 10.1021/acsami.4c19750 (PMC11826499; doi:10.1021/acsami.4c19750)
Supplement: Supplementary file 1 — am4c19750_si_001.pdf [file am4c19750_si_001.pdf]

Supporting Information

Sub-5 nm Silicon Nanopore Sensors: Scalable Fabrication via Self-Limiting Metal-Assisted Chemical Etching

Authors: Fabio De Ferrari[a], Shyamprasad N. Raja[a], Anna Herland[b,c], Frank Niklaus[a], and Göran Stemme[a]**

Affiliations:

[a]Division of Micro and Nanosystems
KTH Royal Institute of Technology
Malvinas väg 10, Stockholm 100 44, Sweden

[b]Division of Nanobiotechnology,
SciLifeLab, Department of Protein Science
KTH Royal Institute of Technology,
Tomtebodavägen 23a, Solna 171 65, Sweden
[c] AIMES - Center for Integrated Medical and Engineering Science,
Department of Neuroscience,
Karolinska Institute, Stockholm 171 77, Sweden.

*Email: shnr@kth.se.
*Email: stemme@kth.se.

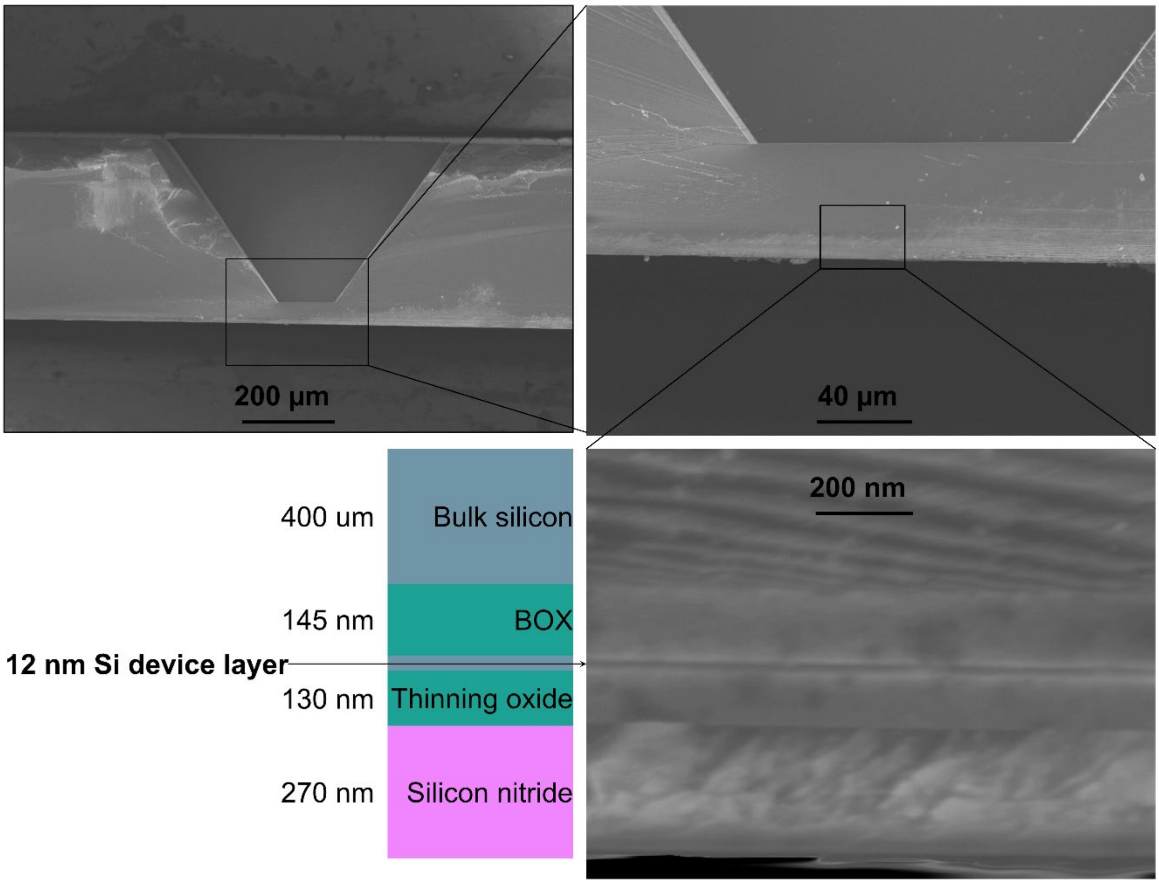

Figure S1 – Scanning electron microscopy (SEM) images of membrane cross-section. a) Membrane with partially etched handle layer of the SOI substrate b) Higher magnification image showing layer stack near the substrate surface. c) Schematic representation of the layer stack of the SOI substrate depicted in d. d) High-magnification SEM image of the Si device layer of the SOI substrate that is sandwiched between the BOX layer and the thermally grown SiO_2 layer.

Supplementary Note 1: Custom protocol for nanoparticle deposition and MACE

Biomolecule detection using single pores provides precise control over signal acquisition and unambiguous event attribution.[1,2] Established fabrication methods typically focus on the serial production of single pores. In our approach we propose parallel fabrication of pores for high manufacturing throughput, in combination with statistical control over the number of nanopores formed per membrane. To develop a suitable nanoparticle deposition and metal-assisted chemical etching (MACE) protocol, we progressed through sequential optimization: (i) initial development on silicon (Si) wafers, followed by (ii) validation on supported silicon-on-insulator (SOI) substrates, and culminating in (iii) implementation on suspended SOI membranes. This hierarchical approach minimized resource utilization while enabling process refinement at each development stage.

We developed a standardized protocol that we used for all samples in this work: (1) 4 µl gold nanoparticles (AuNP) solution deposition, (2) followed by 20 µl 49% hydrofluoric acid (HF) addition, (3) a 30-second particle adhesion period, and (4) subsequent introduction of 4 µl 30% hydrogen peroxide (H_2O_2) to perform MACE (Figure 1a). Surface preparation is critical for deposition yield consistency;[3,4] therefore, we adopted a rigorous cleaning procedure comprising 10 minutes of piranha treatment followed by 10 s of oxide stripping by HF immediately before performing MACE.

Initial gold nanoparticle deposition proceeds through HF-mediated destabilization of the citrate capping.[3] Quantitative analysis of nanoparticle distributions on Si wafers, using a solution concentration of 4.96e12 NPS/ml, revealed significant spatial heterogeneity across multiple regions (Figure S2).

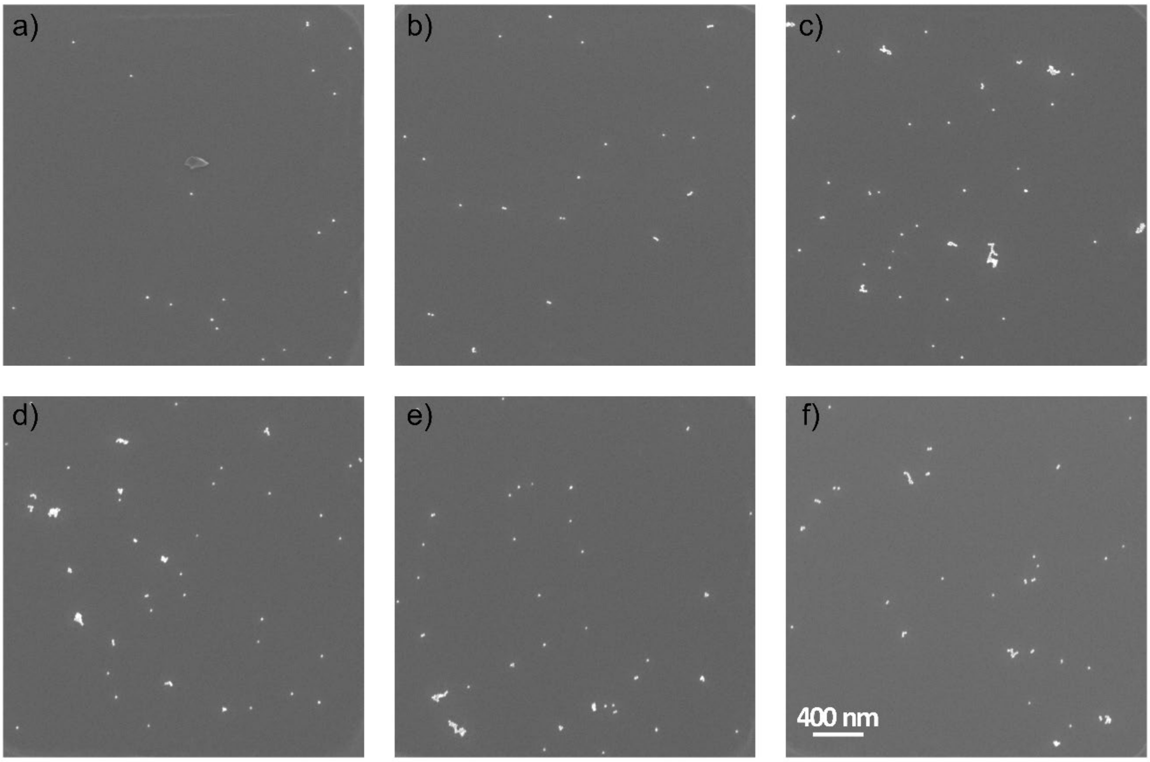

Figure S2 – SEM characterization of Au nanoparticle distribution on a Si substrate. Analysis areas (4.4 µm²) were defined by openings in a silicon nitride coating on the Si susbtrate. Panels (a-c): Sample 1; Panels (d-e): Sample 2. Scale bar consistent across all panels.

Analysis of 4.4 µm² large areas showed that when counting individual particles (whether isolated or in clusters), the mean density was 19.3 ± 11.4 particles/µm² (N=6). However, when analyzing catalytic sites, where each isolated particle and each cluster counts as a single site during MACE, we observed a more consistent mean density of 6.9 ± 1.9 sites/µm² (N=6). This site-based analysis aligns with our TEM observations, where we primarily observed pores formed by individual particles, suggesting our protocol should focus on maximizing the deposition of isolated particles rather than clusters.

To enable controlled wet processing of individual chips, we mounted each 2.5 mm × 2.5 mm chip on protective blue tape commonly used in wafer processing. The tape protected the backside of the chip from HF exposure while providing a convenient handling surface. We performed all liquid processing steps via drop casting directly onto the chip surface, with the tape edges helping contain the liquid. The total processing volume of 28 µl (4 µl Au NPs + 20 µl HF + 4 µl H_2O_2) was optimized to ensure complete coverage of the chip surface while remaining confined within the tape boundaries, preventing coffee ring formation on the chip. Following O'Reilly *et al.*'s protocol,[3] we maintained a short 30-second particle deposition time to minimize aggregation, which affects approximately 15% of particles after 60 seconds.[3]

Both H_2O_2 concentration and MACE duration significantly influence nanopore formation. The volume and concentration of H_2O_2 were selected to maintain a ratio $\rho = [HF]/([HF]+[H_2O_2])$ of approximately 88%, substantially exceeding the critical threshold of 70% necessary for minimizing porous silicon formation and promoting directional etching.[5] The systematic investigation of etching duration revealed that increasing MACE time from 60 s to 120 s resulted in a corresponding increase in nanopore formation, as demonstrated in Figure S3 a-c. However, longer durations of 90 s and 120 s risked compromising membrane integrity due to extensive BOX layer removal, as evidenced by the collapsed Si nanomembranes visible in Figure S3c.

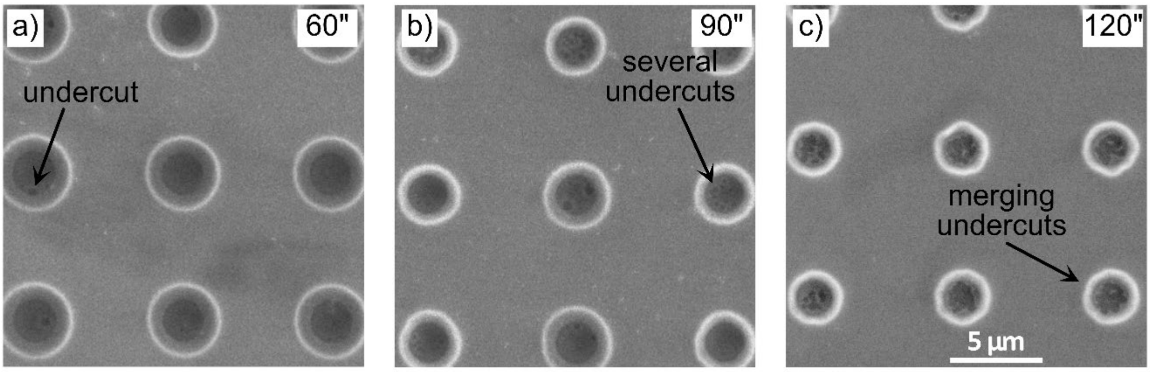

Figure S3 – SEM characterization of MACE-induced features in SOI samples showing representative subsets (9 of 25 total circular openings) after different etching durations. (a) After 60s MACE, sparse undercut formation is observed, with single undercuts (arrow) visible in some openings. (b) At 90s, multiple undercuts develop within individual openings, indicating increased etching progression. (c) Extended etching for 120s leads to extensive undercut merging and membrane destabilization, as evidenced by the significantly higher density of undercuts per opening. Scale bar consistent across all panels.

From our previous particle deposition analysis, we proceeded with the 60s MACE duration, examining four supported SOI samples that demonstrated consistent undercut formation, with an average of

42 ± 9 undercuts per sample (N=4). Each sample contained 25 circular openings, with individual openings showing between zero and seven undercuts. Our statistical analysis revealed that while counting all individual particles (whether isolated or in clusters) might suggest modest conversion rates of 4.5% ± 3.8% (N=4), a more representative assessment based on catalytic sites (where each isolated particle or cluster counts as one site) shows an efficiency of 8.3% ± 2.5% (N=4). This site-based analysis provides more consistent results with lower relative variability and better represents our current process where both individual particles and clusters act as catalytic sites. While our ultimate goal is to achieve single pore formation from individual particles, understanding the role of clusters in our current process is conducive to future protocol optimization.

The process demonstrates reliable device production, with 76% (N=4) of openings containing at least one undercut, resulting in a 100% (N=4) yield of membranes with pores and approximately 42 pores per membrane. It is important to note that to increase the repeatability of this process, we aim to maximize the deposition of individual particles. As mentioned in the main manuscript, solution aging induces particle clustering, and our results indicate that clusters are less likely to form pores during MACE while drastically reducing the effective number of available particles for the reaction to occur. Lastly, it is important to notice that while SEM analysis showed a higher average number of undercuts per sample area than estimated from conductance measurements, this difference can be attributed to incomplete etching and/or non-conducting undercuts in solution. The 60 s duration proved optimal for our application, producing sufficient nanopores for sensing while maintaining minimal pore density across the membrane.

Assuming independent pore formation events with a constant average rate, Poisson distribution analysis indicates that even under ideal conditions, the theoretical maximum probability for single-pore formation per membrane is approximately 37%. This fundamental statistical limit arises from the random nature of the process and cannot be overcome without changing the underlying pore formation mechanism. When targeting single pores, membranes without pores remain in principle viable for subsequent MACE iterations, presenting opportunities for large-scale production utilizing optimized protocols. Thus, it is in principle possible and theoretically attainable to achieve formation of single pores on a large number of membranes. However this requires further protocol optimization, which represents a distinct research challenge that is beyond the scope of the current investigation. Here we prioritized protocol optimization for demonstrating and investigating our nanopore formation approach and to accelerate development iterations.

End of Supplementary Note 1

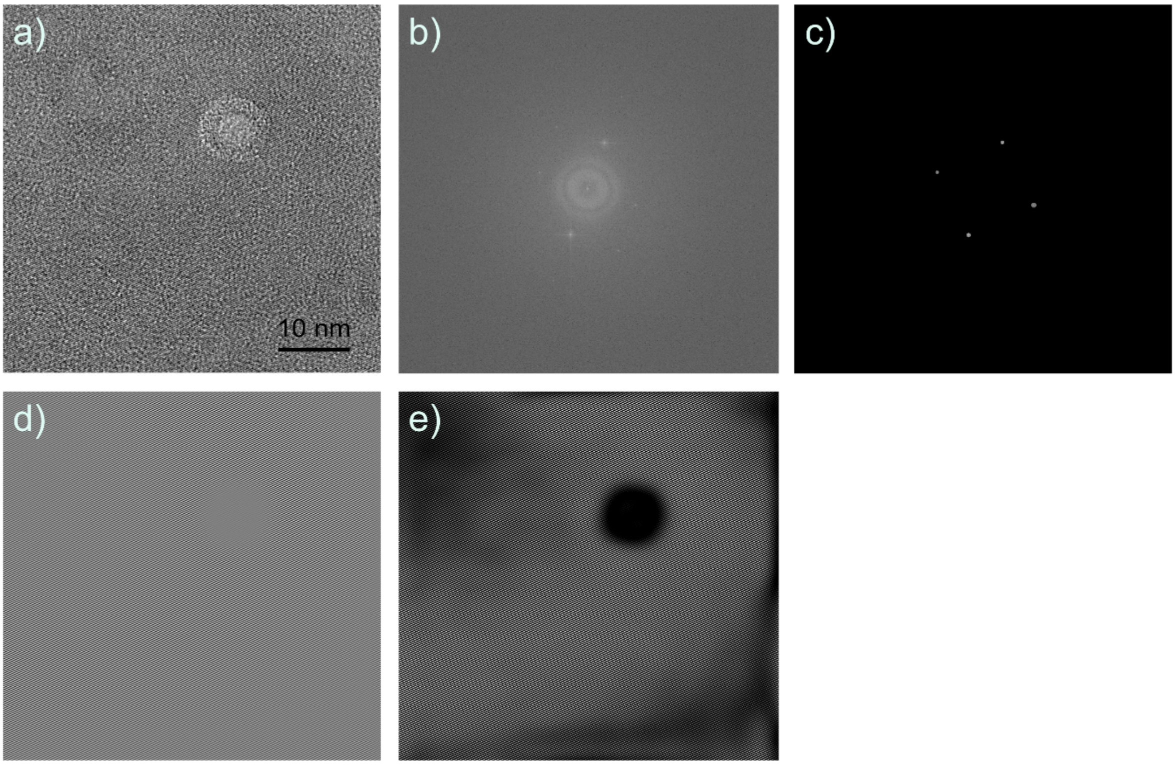

Figure S4 – Identification of crystal planes using TEM images in combination with fast Fourier transform (FFT) for cases when the SiO_2 area is not obvious. a) Original TEM image showing visible crystal planes and the central pore area. b) FFT of the image data. c) Filter selecting only the peaks associated with crystal planes. d) Inverse FFT extracted from the filtered FFT image. e) Brightness and contrast-adjusted image highlighting the area without crystalline Si. Note: This process is used only as a visual guide; measurements were always taken on the original image, as the diameter of the dark area in (e) is highly sensitive to the settings of the image formatting.

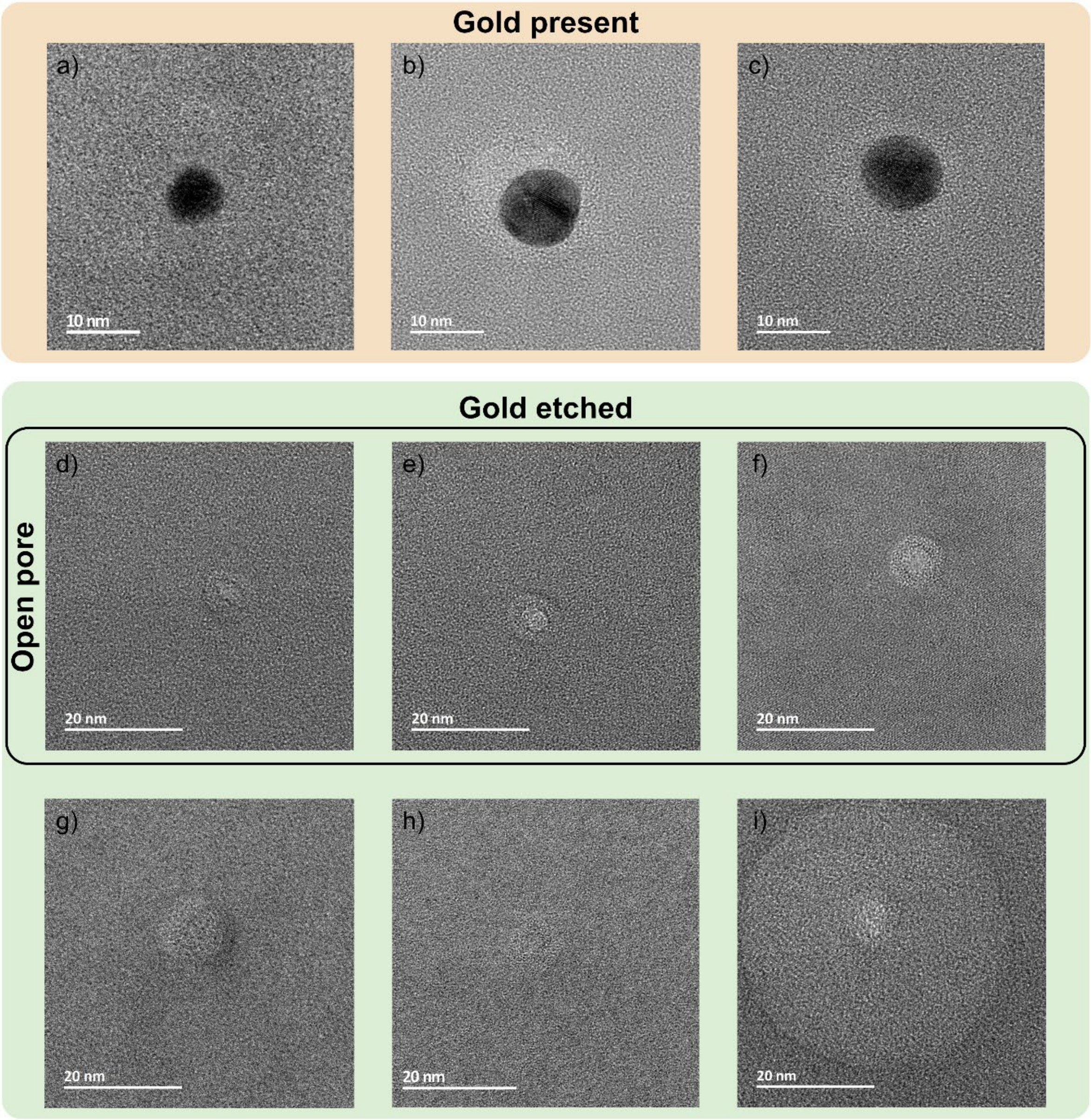

Figure S5 – TEM images. a, b, c) Samples with gold nanoparticles present. In these images, nanopores are present (as evidenced by the etched BOX layer and undercut formation) but are not visible because the gold nanoparticles in front obscure them. In b and c, Si crystal planes are identifiable. d, e, f) Samples where gold was etched after MACE, showing visible pores, Si crystal planes, and oxidized regions. g, h, i) Samples where gold was etched away, but open pore areas were too small to be clearly identifiable.

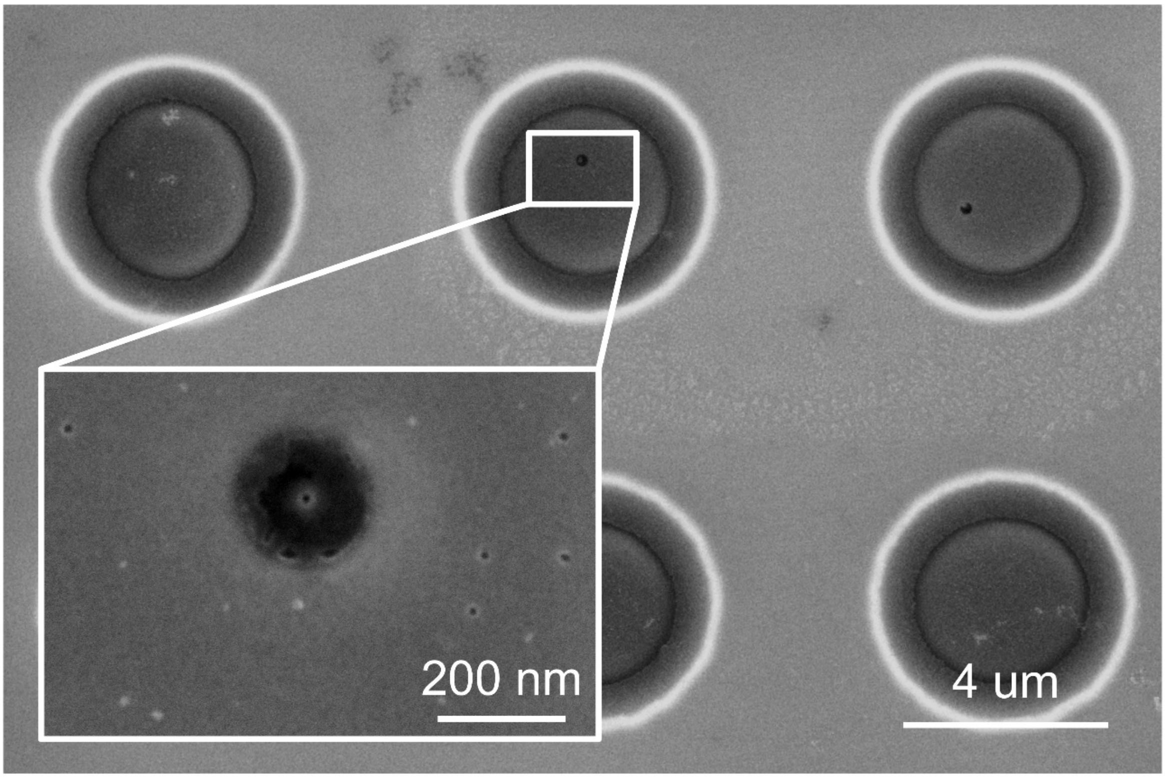

Figure S6 – SEM image of four openings in the top SiN_x layer after MACE. In two of these openings, a single undercut is visible as a small dark circle. These undercuts are used to identify successful nanopore formation and to locate the nanopore. Inset: magnified view of a single undercut and the surrounding supported Si area, showing both a fully formed pore in the center of the undercut and indentations from 10 nm gold nanoparticles that partially etched the supported Si device layer of the SOI substrate before being removed by gold etching prior to imaging.

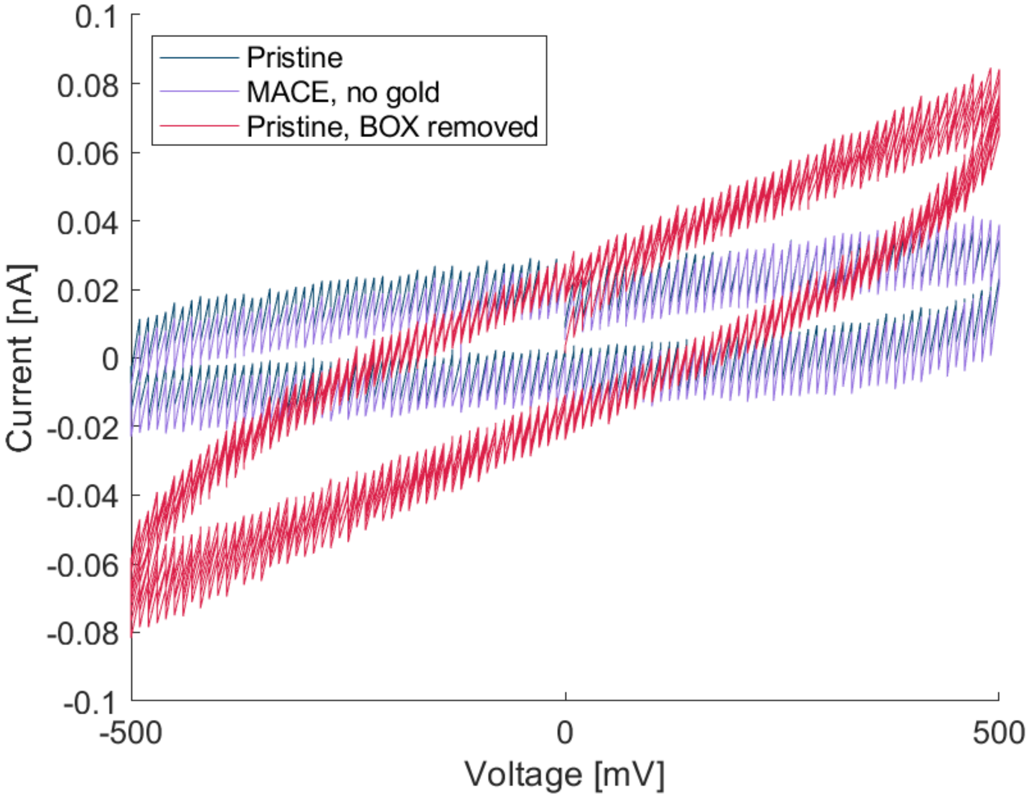

Figure S7 - I-V characterization of reference membranes. Current-voltage sweeps in 1 M KCl recorded from membranes without nanopores: membrane with top SiO_2 removed by HF etching (green), membrane with top SiO_2 removed by MACE without gold nanoparticles (purple), and membrane with both top SiO_2 and BOX layer removed (red). All curves exhibit capacitive behavior with maximum currents below 80 pA, confirming minimal leakage through the Si membrane in absence of nanopores.

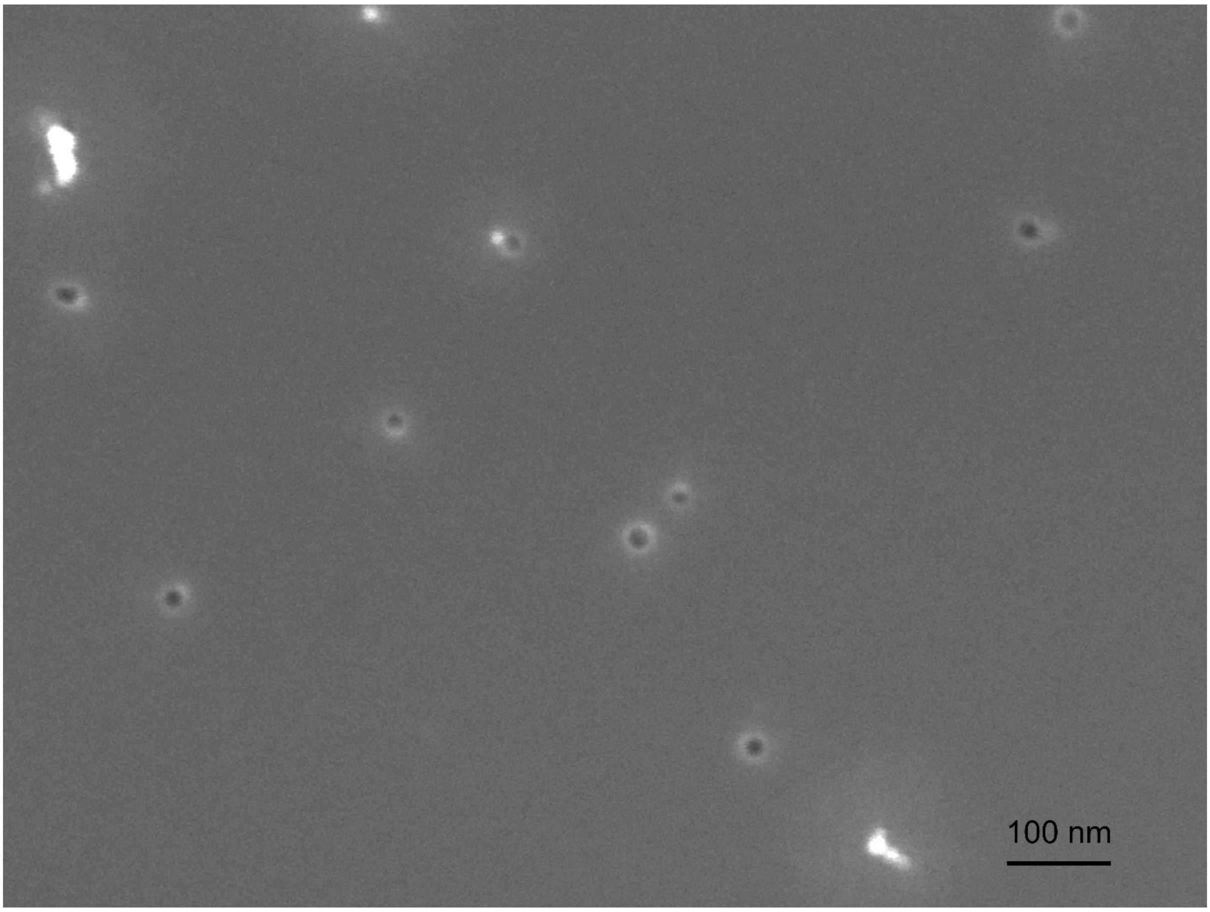

Figure S8 – SEM image of pores formed by the MACE process using 20 nm diameter gold nanoparticles on a bulk Si wafer. The resulting pore diameters are consistently larger than the original gold particle diameters. Note that the gold nanoparticles are buried deep in the Si layer and are not visible in the SEM images. Also, since a bulk Si wafer was used in this experiment, undercut formation does not occur.

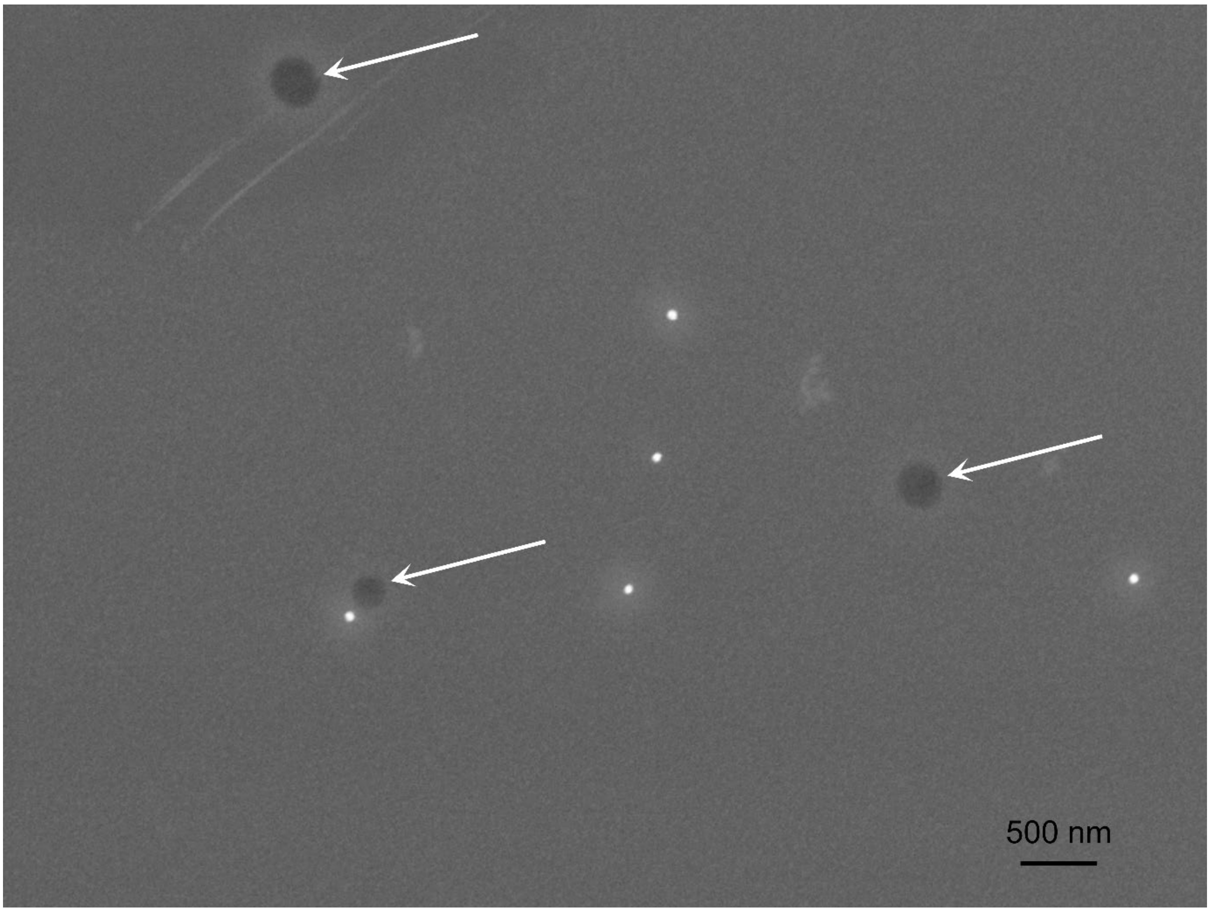

Figure S9 – SEM image of undercuts (white arrows) formed by MACE using 40 nm diameter gold nanoparticles on a plain SOI substrate (12 nm Si device layer, 145 nm BOX layer). The absence of visible pores indicate that the resulting pores feature diameters of below 10 nm.

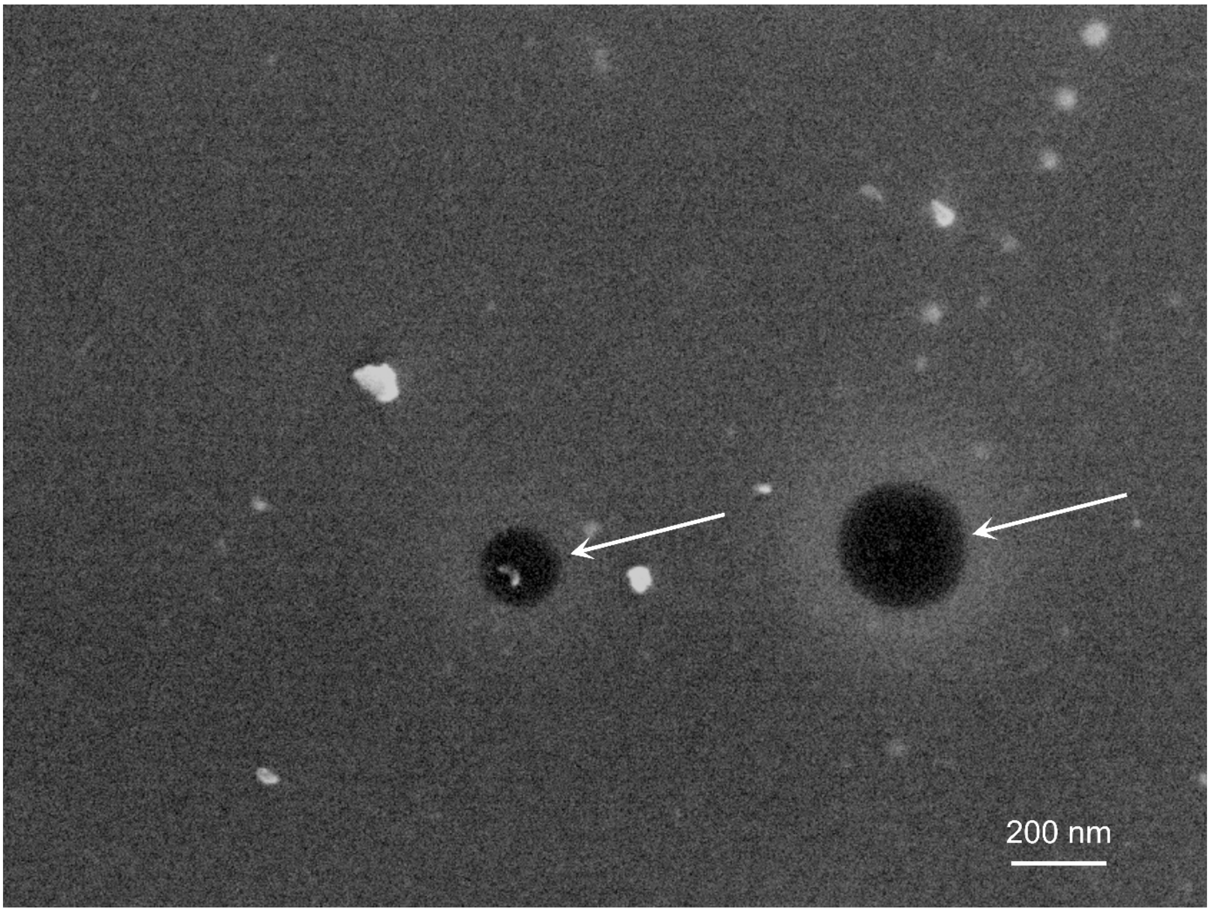

Figure S10 – SEM image of undercuts (white arrows) formed by 100 nm diameter gold nanoparticles on a plain SOI substrate (12 nm Si device layer, 145 nm BOX layer). The absence of visible pores indicate that the resulting pores feature diameters of below 10 nm.

Supplementary Note 2: EF-TEM analysis

Energy-filtered TEM (EF-TEM) was employed to create thickness maps of the nanomembrane area surrounding the nanopores, providing additional insights into nanopore formation and BOX layer etching. This technique offers cross-sectional profile information by computing a relative thickness map (t/λ) from unfiltered and zero-loss images using the Poisson statistics of inelastic scattering: $\frac{t}{\lambda} = -\ln(\frac{I_0}{I_t})$. Here the mean free path (mfp) λ and the thickness t are calculated from the ratio of the total unfiltered intensity I_t and the zero-loss filtered intensity I_0.

Two sample types were examined: one with expected complete BOX removal and another with partial removal. A cross-section including the nanomembrane center, where the nanopore was expected, was extracted from the 2D thickness map at 8kx magnification. In the fully open undercut, the t/λ line profile revealed typical rounded edges of the remaining BOX, formed by isotropic SiO_2 etching. While the pore center didn't show a zero value, likely due to carbon contamination and limited EF-TEM spatial resolution, a local thickness decrease suggested the presence of a sub-10 nm nanopore. A t/λ value of approximately 0.15 was observed surrounding the nanopore, consistent with 12 nm Si and 2 nm SiO2 layers on either side. Away from the center, the t/λ value of 0.7 aligned with the expected presence of the 145 nm thick SiO_2 layer. EF-TEM imaging confirmed significantly higher thickness values in samples with a smaller undercut, indicating residual BOX. This observation reinforces the necessity to perform MACE for a duration sufficient to entirely open the BOX channel while keeping the etching time as short as possible to avoid excessive BOX removal. Although this method can reveal the need for further etching, it proved insufficiently sensitive to confirm complete BOX removal due to limitations in detecting slight thickness variations.

At higher magnification (25kx, Fig. S8), both crystalline patterns indicative of crystalline Si and the nanopore location, marked by decreased thickness, were observed. The pore exhibited a tapered cross-section and did not appear fully open (consistent with previously discussed oxide regrowth and imaging artifacts), further supporting the partial penetration model.

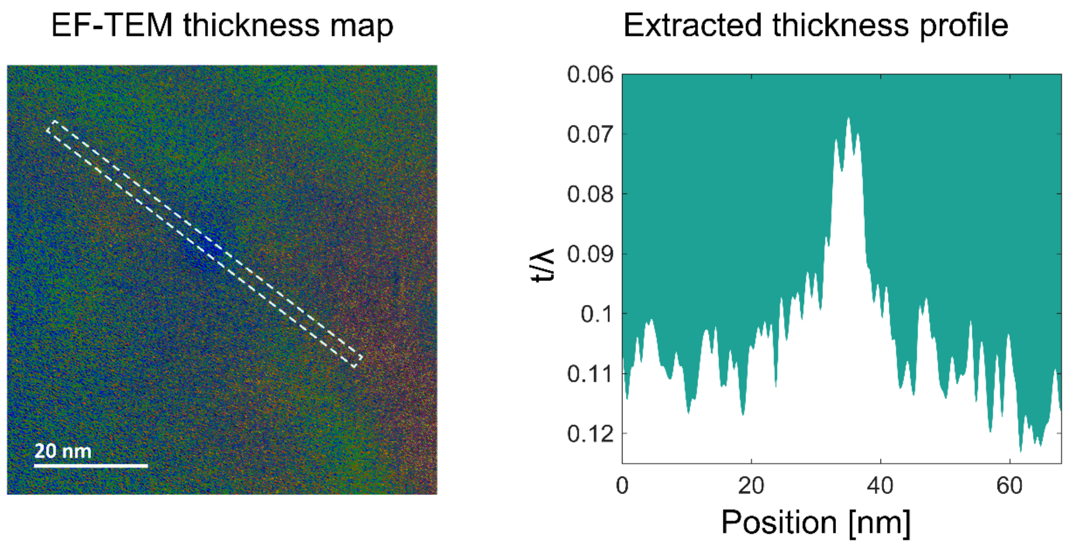

Figure S11 – EF-TEM thickness map (left) at 25 000x magnification with a 30-pixel wide selection window. Extracted thickness profile from the window (right), demonstrating the presence of a sub-10 nm diameter nanopore.

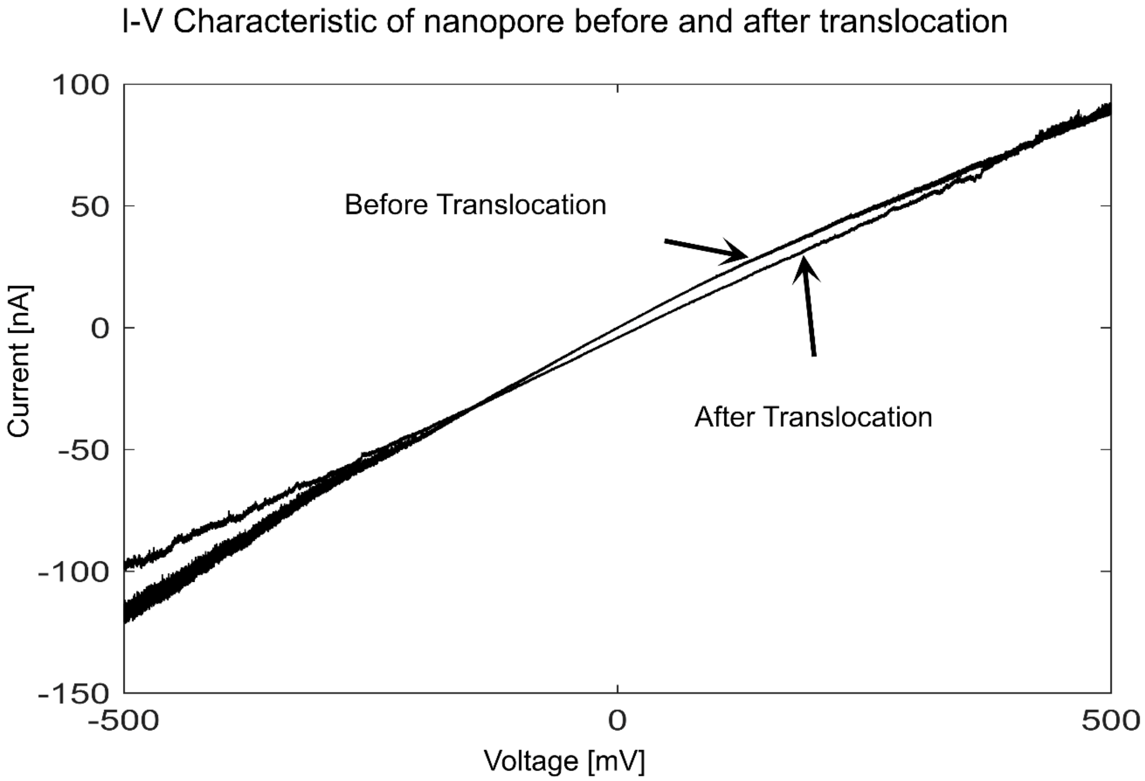

Figure S12 – I-V curves from a nanopore sensor device with 210 nS conductance during electrical characterization. Curve fitting immediately before DNA translocation experiments showed a conductance of 206.0 nS ± 0.1 nS, which decreased slightly to 189.0 nS ± 0.1nS after six hours of DNA translocation experiments.

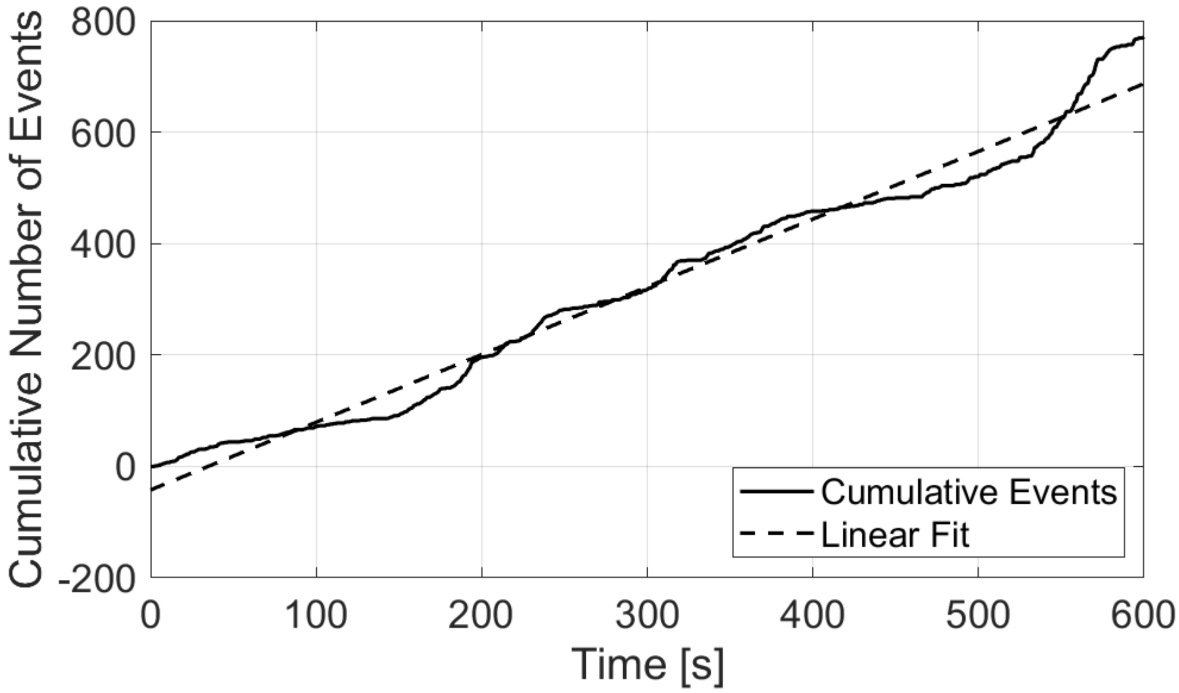

Figure S13 – Cumulative count of translocation events over time.

Supplementary References

(1) Drndić, M. 20 Years of Solid-State Nanopores. *Nature Reviews Physics 2021 3:9* **2021**, *3* (9), 606–606. https://doi.org/10.1038/s42254-021-00363-w.

(2) Xue, L.; Yamazaki, H.; Ren, R.; Wanunu, M.; Ivanov, A. P.; Edel, J. B. Solid-State Nanopore Sensors. *Nat Rev Mater* **2020**, 931–951. https://doi.org/10.1038/s41578-020-0229-6.

(3) O'Reilly, A. J.; Francis, C.; Quitoriano, N. J. Gold Nanoparticle Deposition on Si by Destabilising Gold Colloid with HF. *J Colloid Interface Sci* **2012**, *370* (1), 46–50. https://doi.org/10.1016/j.jcis.2011.12.012.

(4) Kong, L.; Zhao, Y.; Dasgupta, B.; Ren, Y.; Hippalgaonkar, K.; Li, X.; Chim, W. K.; Chiam, S. Y. Minimizing Isolate Catalyst Motion in Metal-Assisted Chemical Etching for Deep Trenching of Silicon Nanohole Array. *ACS Appl Mater Interfaces* **2017**, *9* (24), 20981–20990. https://doi.org/10.1021/acsami.7b04565.

(5) Huang, Z.; Geyer, N.; Werner, P.; de Boor, J.; Gösele, U. Metal-Assisted Chemical Etching of Silicon: A Review. *Advanced Materials* **2011**, *23* (2), 285–308. https://doi.org/10.1002/adma.201001784.
